# A Biomimetic Approach to Premyrsinane-Type Diterpenoids: Exploring Microbial Transformation to Enhance Their Chemical Diversity

**DOI:** 10.3390/plants13060842

**Published:** 2024-03-14

**Authors:** Felipe Escobar-Montaño, Antonio J. Macías-Sánchez, José M. Botubol-Ares, Rosa Durán-Patrón, Rosario Hernández-Galán

**Affiliations:** 1 Departamento de Química Orgánica, Facultad de Ciencias, Universidad de Cádiz, Puerto Real, 11510 Cádiz, Spain; felipe.escobarmonta@gm.uca.es (F.E.-M.); antoniojose.macias@uca.es (A.J.M.-S.); rosario.hernandez@uca.es (R.H.-G.); 2 Instituto Universitario de Investigación en Biomoléculas, Universidad de Cádiz, Puerto Real, 11510 Cádiz, Spain; 3 Instituto Universitario de Investigación Vitivinícola y Agroalimentaria, Universidad de Cádiz, Puerto Real, 11510 Cádiz, Spain

**Keywords:** *Euphorbia boetica*, 6,17-epoxylathyrane, epoxyboetirane A, premyrsinane, titanocene, transannular cyclization, *Mucor circinelloides*, biotransformation

## Abstract

Premyrsinane-type diterpenoids have been considered to originate from the cyclization of a suitable 5,6- or 6,17-epoxylathyrane precursor. Their biological activities have not been sufficiently explored to date, so the development of synthetic or microbial approaches for the preparation of new derivatives would be desirable. Epoxyboetirane A (**4**) is an 6,17-epoxylathyrane isolated from *Euphorbia boetica* in a large enough amount to be used in semi-synthesis. Transannular cyclization of **4** mediated by Cp_2_Ti^III^Cl afforded premyrsinane **5** in good yield as an only diasteroisomer. To enhance the structural diversity of premyrsinanes so their potential use in neurodegenerative disorders could be explored, compound **5** was biotransformed by *Mucor circinelloides* NRRL3631 to give rise to hydroxylated derivatives at non-activated carbons (**6**–**7**), all of which were reported here for the first time. The structures and absolute configurations of all compounds were determined through extensive NMR and HRESIMS spectroscopic studies.

## 1. Introduction

The *Euphorbia* genus is the largest of the Euphorbiaceae family; it contains more than 2000 species, and has a widespread distribution and a high chemical diversity [1,2,3,4,5]. A great number of polycyclic and macrocyclic diterpenoids with a broad range of therapeutically relevant biological activities have been isolated from *Euphorbia* species including jatrophane [6], tiglianes [7], ingenanes [8], and lathyranes [9].

Premyrsinanes-type diterpenoids have been isolated from approximately 20 *Euphorbia* species and *Jatropha jurcus* [10]. They are characterized by a 5/7/6/3-tetracyclic carbon framework with *trans*-oriented A/B and B/C ring junctions. As described in several reports, they often exhibit three characteristic oxymethyne protons at C-3, C-5, and C-7 positions and one oxygenated methylene group at C-17 [10,11].

**Scheme 1:**
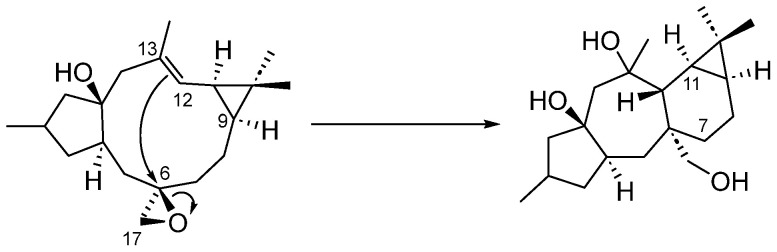
Biogenetic relationship between lathyranes and premyrsinanes.

**Scheme 2:**
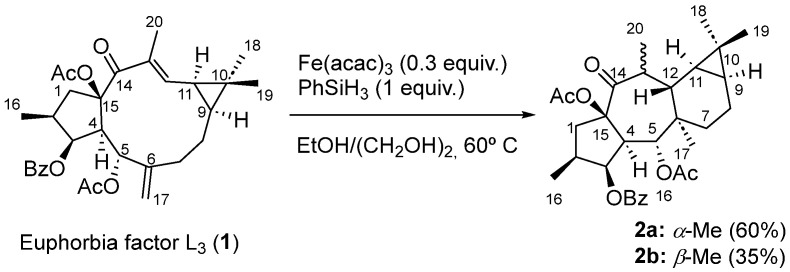
Chemical conversion of lathyradiene **1** into premyrsinanes **2a** and **2b** mediated by Fe.

From a biogenetic point of view, premyrsinanes could be derived from a lathyrane precursor by intramolecular cyclization between the C-12 and C-6 positions. However, their biogenetic relationships still remain unclear. For instance, 5,6- and 6–17-epoxylathyranes could be considered biosynthetic precursors of premyrsinanes [12,13]. Appendino et al. proposed a biogenetic relationship between lathyranes and premyrsinanes based on the co-occurrence of these diterpenoids with a similar functionalization pattern (Scheme 1). They suggested that the conversion of a suitable 6,17-epoxylathyrane would lead to premyrsinane derivative through a transannular cyclization but so far it has not been achieved using synthetic approaches [14]. Recently, Xiao et al. performed the chemical conversion, catalyzed by Fe(acac)_3_, of Euphorbia factor L_3_, a Δ^6(17),12^ lathyradiene (**1**), into premyrsinane skeleton compounds. The reaction involved an intramolecular Michael addition via free radicals to produce a mixture of diasteroisomeric premyrsinanes **2a**–**b** [15,16] (Scheme 2). 

Studies of the biological activities of premyrsinanes are scarce in the literature; this is likely because of the reduced amount of the isolated compounds [10]. Nevertheless, several studies have reported that premyrsinanes possess promising anticancer [17,18,19], multidrug resistance reversal [20], and anti-inflammatory activities, inhibiting the lipopolysaccharide-induced NO production [21]. Furthermore, their potential biosynthetic precursors, euphoboetirane A (**3**) and epoxyboetirane A (**4**), have demonstrated a promising activity as promoters of neural stem cells (NSC) proliferation [22]. For this reason, it is necessary to develop synthetic or microbial methodologies to obtain new premyrsinane derivatives in order to explore its biological activity and to study structure–activity relationships.

Cp_2_Ti^III^Cl has emerged as a useful reagent in organic chemistry because of its versatility to generate single-electron-transfer reactions [23,24,25,26,27]. This complex is easily prepared from commercially available Cp_2_Ti^IV^Cl_2_ by reduction with metals such as Zn [28] or Mn [29]. The reaction of Cp_2_Ti^III^Cl with oxiranes produces its homolytic cleavage to afford a *β*-titanoxyl radical [30]. This radical can also react with activated olefins to form carbon-carbon bonds [31]. Consequently, we postulate that the titanium-catalyzed cyclization of 6,17-epoxylathyranes could be a powerful tool for the preparation of premyrsinane derivatives.

On the other hand, microbial transformations have advantages over conventional organic synthesis due to their high stereo- and regioselectivity under mild reaction conditions [32,33,34,35]. In the literature, there are few examples of microbial transformations of lathyrane-type compounds involving regioselective hydroxylations, glycosylations, deoxygenations, and cyclopropane ring openings [36,37,38]. Recently, we reported the microbial transformation of euphoboetirane A (**3**) by *Streptomyces puniceus* BC-5GB.11; this resulted in regioselective oxidation of non-activated carbons, isomerization of the Δ^12^ double bond, and cyclopropane rearrangements [39]. To the best of our knowledge, this strategy has not been used for the functionalization of non-activated positions of premyrsinane-type diterpenoids.

Therefore, as a part of our ongoing research on compounds with diterpene scaffold for the treatment of neurodegenerative disorders, we describe here an unprecedented titanium-catalyzed cyclization of a 6,17-epoxylathyrane isolated from *Euphorbia boetica*, epoxyboetirane A (**4**) [22,40], to premyrsinane **5**. Furthermore, we also describe the biotransformation of compound **5** by *Mucor circinelloides* NRRL3631 in order to increase the structural diversity of premyrsinane-type diterpenoids.

## 2. Results and Discussion

**Scheme 3:**
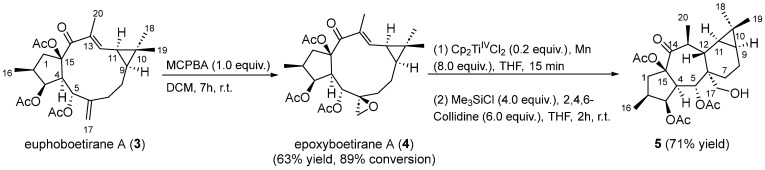
Preparation of premyrsinane **5**.

Euphoboetirane A (**3**) [41] and epoxyboetirane A (**4**) [40] were isolated from the aerial parts of *E. boetica*, an endemic plant of the southern Iberian Peninsula, collected from “El Pinar del Hierro” in Chiclana de la Frontera, Cádiz (Spain). These lathyranes were purified by column chromatography from the *n*-hexane-soluble fraction of the methanolic extract of the plant, following the procedure reported previously in the literature [39]. In order to confirm its proposed absolute stereochemistry, compound **4** was synthesized by treatment of euphoboetirane A (**3**) [41] with MCPBA (Scheme 3). ^1^H NMR data for isolated and synthetic compound **4** were found to be coincident (Figure S2). Since the absolute configuration of **3** has been described as 2*S*,3*S*,4*R*,5*R*,6*S*,9*S*,11*R*,15*R*, based on X-ray and ECD studies [41,42,43,44], the chemical conversion of euphoboetirane A (**3**) into **4** allow us to corroborate the proposed absolute configuration of (2*S*,3*S*,4*R*,5*R*,6*S*,9*S*,11*R*,15*R*)-epoxyboetirane A (**4**) [40].

Encouraged by the postulated biogenetic relationship between 6,17-epoxylathyranes and premyrsinanes [14], we explored a transannular cyclization of **4** catalyzed by Cp_2_Ti^III^Cl. This reagent was generated by reduction of catalytic quantities of Cp_2_Ti^IV^Cl_2_ (0.2 equiv.) with excess of Mn powder (8 equiv.) in deoxygenated and dry THF in the presence of 2,4,6-collidine and TMSCl [45]. After 15 min, the mixture turned to the characteristic lime green color of Ti(III) solutions. Gratifyingly, premyrsinane **5** was isolated in good yield after 2 h as a result of a 6-endo-trig intramolecular cyclization. Interestingly, the reaction occurred with high regio- and diastereoselectivity (Scheme 3). Contrary to the Fe-mediated cyclization of Euphorbia factor L_3_ [21], premyrsinane **5** was obtained at room temperature as only one diastereoisomer, with the same stereochemistry at C-6, C-12, and C-13 than most naturally occurring premyrsinane-type diterpenoids [10]. Our newly developed protocol would represent the first report of a biomimetic cyclization of a 6,17-epoxylathyrane into premyrsinane derivative.

**Figure 1:**
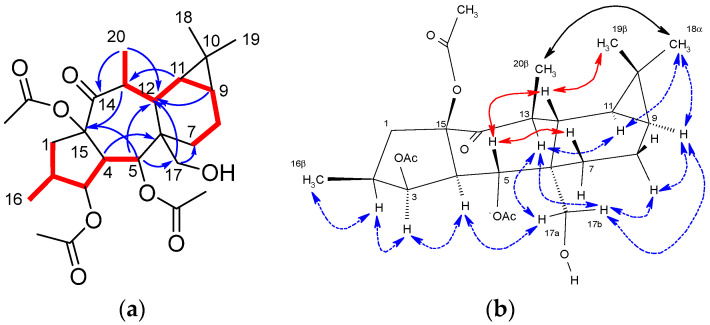
(**a**) Key COSY (red bond) and HMBC (blue arrows) correlations for compound **5**. (**b**) Selected 1D and 2D NOESY correlations for compound **5**. *β*-Face correlations in red. *α*-Face correlations in blue. Correlations involving nuclei in α-face and *β*-face in black.

Compared with the starting material (compound **4**), the 12,13-double bond and 13-vinylic methyl group characteristic signals were absent in the NMR spectra of compound **5** (Figures S3–S9) [40]. Furthermore, resonances which could be attributed to a hydroxymethyl group (*δ*_H_ 3.84 (d) and 3.68 (d); *δ*_C_ 63.2) and a methyl attached to a methyne (*δ*_H_ 1.10 (d); *δ*_C_ 19.7; H_3_-20) were present in the NMR spectra of **5**, which could be located at C-6 and C-13, respectively, on a premyrsinane skeleton (Table 1). Analysis of the ^1^H-^1^H COSY spectrum (Figure S5) revealed two spin systems: H_2_-1/H-2/H_3_-16/H-3/H-4/H-5 and H_3_-20/H-13/H-12/H-11/H-9/H_2_-8/H_2_-7. Moreover, HMBC correlations (Figure S7) from H_2_-17 to C-6 and C-12, H_3_-20 to C-12, C-13 and C-14, and H-5 to C-6 and C-12 were observed, consistent with a 5/7/6/3-fused tetracyclic ring skeleton for **5**, characteristic of premyrsinane derivatives. 1D and 2D NOESY correlations (Figures S8 and S9) between H-2/H-3/H-4/H_2_-17/H-8*α*/H-9/H_3_-18/H-11/H-13 and H_2_-17/H-13 indicated that they all are *α*-oriented, while correlations between H-5/H-12/H_3_-19 and H-5/H-7*β* supported their *β*-orientation (Figure 1). 

Based on the above-mentioned NOESY correlations and the absolute configuration described above for epoxyboetirane A (**4**), the structure of **5** was determined to be (2*S*,3*S*,4*R*,5*R*,6*R*,9*S*,11*S*,12*S*,13*S*,15*R*)-3,5,15-triacetoxy-7,13-dideoxipremyrsinol. 

**Scheme 4:**
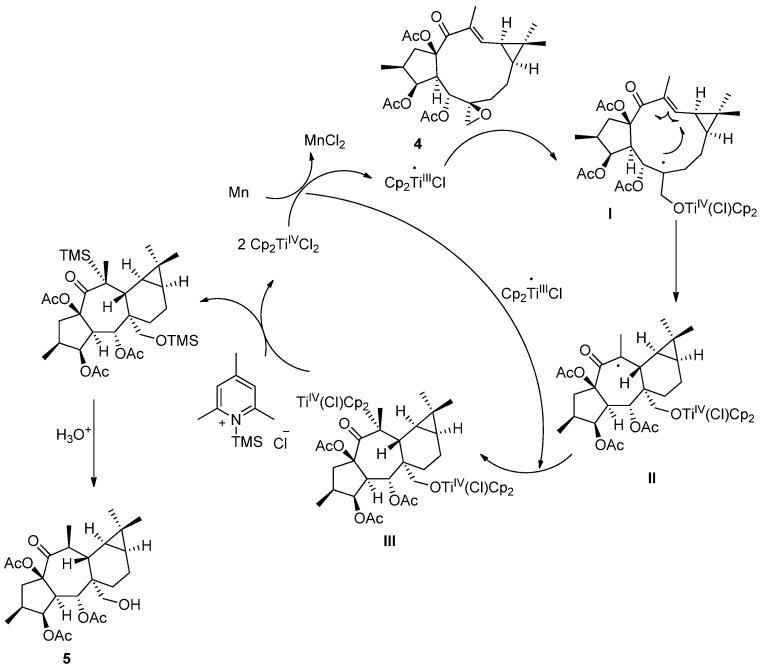
Catalytic cycle for the Ti(III)-mediated radical transannular cyclization.

In accordance with the mechanism admitted for catalytic cyclization mediated by Cp_2_Ti^III^Cl, a catalytic cycle is proposed in Scheme 4 to rationalize the formation of **5** [45]. The homolytic opening of the epoxide leads to a *β*-titanoxyl radical (**I**), which is trapped by the electron deficient 12,13 double bond in **4** [46]. The control of the observed diastereoselectivity in the cyclization step could be explained on the basis of the 14d20d preferred conformation for **4** (Figure S1). This conformation would be consistent with a Ti-carbonyl coordination [47], favoring an approach of the radical to the double bond leading to a *trans*-fused ring. Then, the carbon-centered radical resulting from the cyclization step is trapped by a second molecule of Cp_2_Ti^III^Cl to produce the alkyltitanium species **III**. Ti-carbonyl coordination would be again essential to explain the *β*-orientation of the methyl group at C-20. Finally, the 2,4,6-trimethylsilylpyridinium chloride obtained by mixing 2,4,6-collidine and TMSCl is capable of regenerating Cp_2_Ti^IV^Cl_2_ and finally yields compound **4**.

**Scheme 5:**
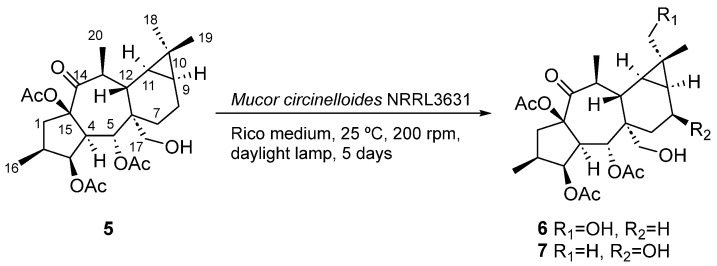
Biotransformation of **5** by *Mucor circinelloides* NRRL3631.

In order to enrich the chemical diversity of premyrsinane-type diterpenoids, compound **5** was subjected to biotransformation. Initial screenings were performed to determine the optimal microorganism and culture conditions. Based on previous experiments of euphoboetirane A (**3**) with about 20 microorganisms (fungi and bacteria), *Mucor circinelloides* NRRL3631 was selected for its ability to biotransform macrocyclic diterpenes [48]. A scaled-up biotransformation of **5** involved feeding of a 3-day-old culture of *M. circinelloides* with **5** and incubation for further 5 days. Two closely related premyrsinane derivatives were obtained: compounds **6** (29% conversion) and **7** (45% conversion), as a result of the regioselective hydroxylation at non-activated positions (Scheme 5). 

**Figure 2:**
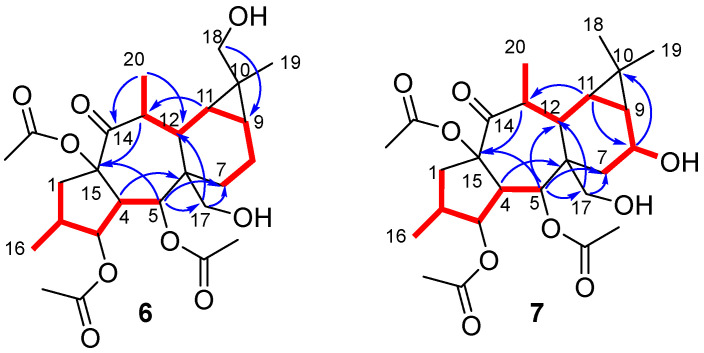
Key COSY (red bond) and HMBC (blue arrows) correlations for compounds **6** and **7**.

Compounds **6** and **7** showed a molecular formula C_26_H_38_O_9_, deduced from [M + Na]^+^ molecular ions in their HRMS spectra (*m/z* 517.2401 and 517.2405, respectively, calculated for C_26_H_38_O_9_Na 517.2414) (Figures S19 and S28). The NMR spectra of **6** (Figures S12–S18) showed an oxygenated methylene carbon signal at *δ*_C_ 72.8, which was correlated in the HSQC spectrum (Figure S15) to two doublet signals at *δ*_H_ 3.41 and 3.30 (Table 1). The hydroxyl group was located at C-18 based on HMBC and NOESY experiments (Figures S16–S18). Thereby, HMBC correlations from *δ*_H_ 1.11 (H_3_-19), 0.34 (H-11) and 0.85 (H-9) to *δ*_C_ 72.8 (C-18), along with NOESY correlations H_2_-18/H-9/H-11/H-8α confirmed the hydroxylation at C-18 (Figure 2). Accordingly, the structure of **6** was identified as (2*S*,3*S*,4*R*,5*R*,6*R*,9*S*,10R,11*S*,*12S*,13*S*,15*R*)-3,5,15-triacetoxy-7,13-dideoxi-18-hydroxypremyrsinol.

On the other hand, the ^1^H NMR spectrum of compound **7** (Figure S21) showed a double of triplet signal at *δ*_H_ 4.37, which was correlated in the gHSQC spectrum (Figure S24) to an oxygenated methine carbon at *δ*_C_ 64.9 (Table 1). These data suggested the presence of a hydroxyl group, which was located at C-8 based on HMBC correlations (Figure 2 and Figure S24) from H-8 to C-10 and from H-11 to C-8, as well as analysis of ^1^H-^1^H COSY correlations (Figure 2 and Figure S23). 1D and 2D NOESY spectra (Figures S26 and S27) showed correlations for H-8/H-7*α*/H-9, H-11/H-9/H_2_-18/H_3_-20/H-13 and H_3_-19/H-12/H-7*β*, supporting the assignment of a *β*-stereochemistry to the hydroxyl group at C-8. Consequently, the structure for **7** was proposed as (2*S*,3*S*,4*R*,5*R*,6*R*,8*S*,9*S*,11*R*,12*S*,13*S*,15*R*)-3,5,15-triacetoxy-7,13-dideoxi-8-hydroxypremyrsinol.

## 3. Materials and Methods

### 3.1. General Experimental Procedures

All air-sensitive manipulations were conducted employing standard Schlenk techniques, under an argon atmosphere and utilizing oven-dried glassware. Tetrahydrofuran (THF) was freshly distilled from Na-benzophenone. Optical rotations were measured using a JASCO P-2000 polarimeter (JASCO, Tokyo, Japan). IR spectra were recorded on a PerkinElmer Spectrum BX FT-IR spectrophotometer (PerkinElmer, Waltham, CA, USA) and are reported in frequency of absorption (cm^−1^). ECD and UV spectra were measured in methanol solutions with a JASCO J-1500 CD spectrometer (JASCO, Tokyo, Japan). NMR spectra were carried out on a Bruker 400 NMR spectrometer (Billerica, MA, USA), using CDCl_3_ as solvent (Eurisotop, Saint-Aubiu, France). Chemical shifts are expressed in ppm (*δ*) referenced to the solvent (*δ*_H_ 7.25, *δ*_C_ 77.0). 2D NMR experiments were performed using a standard Bruker pulse sequence. High-resolution mass spectrometry (HRMS) was obtained on a QTOF mass spectrometer (Xevo-G2-S QTOF; Waters, Manchester, UK) in the positive-ion ESI mode. TLC was performed on Merck Kiesegel Å F_254_, 0.25 mm layer thickness. Column chromatography was carried out on silica gel 60 (60−200 µm, VWR). Purification by HPLC was performed on a Merck–Hitachi Primade apparatus equipped with a UV−vis detector (Primaide 1410) and a refractive index detector (RI-5450), and on a Merck–Hitachi LaChrom apparatus equipped with a UV−vis detector (L 4250) and a differential refractometer detector (RI-7490) (Merck, Darmstadt, Germany). LiChroCART LiChrospher Si 60 (5 μm, 250 mm × 4 mm), LiChroCART LiChrospher Si 60 (10 μm, 250 mm × 10 mm), and ACE 5 SIL (250 mm × 4.6 mm id) columns were used for isolation experiments. 

### 3.2. Plant Material

*E. boetica* was collected in March 2020 from El Pinar del Hierro (Chiclana de la Frontera), Cádiz, Spain, under the authorization of the competent national authorities (reference numbers ESNC64 and 201999901092011).

### 3.3. Microorganism

*M. circinelloides* NRRL3631 was kindly provided by Prof. Cerdá-Olmedo of the University of Sevilla (Spain). Conidia of this strain were preserved in 80% glycerol at −40 °C.

### 3.4. Extraction and Isolation of Euphoboetirane A (***3***) and Epoxyboetirane A (***4***)

The aerial parts of fresh *E. boetica* plant (3.0 kg) were frozen with liquid nitrogen, ground into powder, and subsequently extracted with MeOH (2.5 L × 3) at room temperature for 24 h. After solvent removal, the crude extract was suspended in water (1 L) and extracted with hexane (1.5 L × 3). The obtained crude extract (33.3 g) was subjected to column chromatography on silica gel, using an increasing gradient of EtOAc in *n*-hexane (10–100%) as the mobile phase. Fractions containing lathyranes were further purified by column chromatography, employing an increasing gradient of CH_2_Cl_2_ in acetone (0–2%) to give 2.0 g of euphoboetirane A (**3**) and 345.0 mg of epoxyboetirane A (**4**) [38]. 

### 3.5. Preparation of Epoxyboetirane A (***4***) by Epoxidation of Euphoboetirane A (***3***)

A solution of euphoboetirane A (**3**) (43.1 mg, 0.1 mmol) in CH_2_Cl_2_ (4 mL) was treated with *m*-chloroperbenzoic acid (21.0 mg, 0.1 mmol, 77% *w/w*) and stirred at room temperature for 7 h. Then, a solution of saturated NaHCO_3_ was added and the mixture was extracted with CH_2_Cl_2_ (×3). The organic phase was washed with H_2_O and dried over anhydrous Na_2_SO_4_. Subsequently, the solvent was evaporated using a rotary evaporator, and the resulting reaction mixture was subjected to column chromatography to give 12.3 mg of euphoboetiranne A (**3**) and 28.3 mg of epoxyboetirane A (**4**) (63% yield, 89% conversion). The reaction was repeated several times, with progressively longer reaction times, in an attempt to enhance conversion. In all cases, unreacted starting material was observed not improving yield and conversion stated above.

### 3.6. Preparation of Premyrsinane ***5*** by Cyclization of Epoxyboetirane A (***4***) with Cp_2_Ti^III^Cl

A mixture of Cp_2_Ti^IV^Cl_2_ (10.0 mg; 0.04 mmol; 0.2 equiv.) and Mn dust (39.4 mg, 1.6 mmol, 8 equiv.) in strictly deoxygenated and dry THF (2.8 mL) was stirred at room temperature under an argon atmosphere for 15 min until it turned lime green. Then, a solution of epoxyboetirane A (**4**) (135.0 mg, 0.2 mmol) in dry THF (0.6 mL), 2,4,6-collidine (0.16 mL, 1.2 mmol, 6 equiv.), and Me_3_SiCl (0.10 mL, 0.8 mmol, 4 equiv.) were sequentially added and the mixture was stirred for 2 h. The reaction was diluted with EtOAc (15 mL) and washed with 1M HCl (3 × 5 mL). The organic layer was dried with anhydrous Na_2_SO_4_. Solvent was removed under reduced pressure to give a crude, which was purified by silica gel column chromatography using as eluent *n*-hexane:EtOAc (75:25) to afford compound **5** (95.9 mg, 71% yield).

(2S,3S,4R,5R,6R,9S,11S,12S,13S,15R)*-3,5,15-Triacetoxy-7,13-dideoxipremyrsinol* (**5**). White amorphous powder;$\;{\left[\alpha \right]}_{D\;}^{21}$ = −30.3 (c 3.1, MeOH); ^1^H and ^13^C NMR data, see Table 1 and Figures S3 and S4; gHMBC (selected correlations) H-3 → C-1, C-15, OCO-3; H-4 → C-14, C-5, C-6; H-5 → C-3, C-4, C-6, C-7, C-12, C-15, C-17, OCO-5; H-9 → C-7, C-12, C-19; H-11 → C-9, C-10, C-13, C-18, C-19; H-13 → C-11, C-12, C-15, C-20; H_2_-17 → C-5, C-6, C-7, C-12; H_3_-20 → C-12, C-13, C-14; OCOMe-3 → C-3, OCO-3; OCOMe-5 → C-5, OCO-5; OCOMe-15 → C-15, OCO-15 (Figure S7); IR (film) *υ*_max_ 3513, 2941, 1739, 1372, 1239, 1024 cm^−1^; UV (MeOH) *λ*_max_ (log *ɛ*) 301 (−0.58) nm; ECD (MeOH) *λ* (Δ*ε*) 204 (1.90), 220 (−0.84), 290 (−0.52) nm (Figure S11); HRESIMS [M + Na]^+^ *m/z* calcd for C_26_H_38_O_8_Na, 501.2464; found 515.2467 (Figure S10).

### 3.7. Biotransformation of Premyrsinane ***5*** by Mucor circinelloides NRRL3631

*M. circinelloides* NRRL3631 was cultured in five 500 mL-Erlenmeyer flasks containing 200 mL of Rico medium, consisting of 20.0 g glucose and 1.0 g yeast extract in 500 mL of distilled water; 2.0 g l-asparagin, 5.0 g KH_2_PO_4_, 0.5 g MgSO_4_⋅7H_2_O in 480 mL of distilled water; 10 mL sutter solution and 10 mL calcium sutter solution. The sutter solution contained 0.01% thiamine, 2.0% citric acid, 0.015% Fe(NO_3_)_3_⋅9H_2_O, 0.01% ZnSO_4_⋅7H_2_O, 0.003% MnSO_4_⋅H_2_O, 0.0005% CuSO_4_⋅5H_2_O, and 0.0005% Na_2_MoO_4_⋅2H_2_O. The calcium sutter solution additionally contained 0.28% CaCl_2_. Each flask was inoculated with 0.5 × 10^7^ fresh conidia and incubated for 3 days at 25 °C and 200 rpm under white light (daylight lamp). Then, 300 µL of a DMSO solution containing compound **5** was added to each flask, achieving a final concentration of 70 ppm. Compound **5** was prepared by cyclization of epoxyboetirane A (**4**) isolated from *E. boetica* as described in Section 3.6. Controls for both substrate and culture were prepared. The culture control consisted of a fermentation flask containing 300 µL of DMSO. The substrate control was prepared by adding a solution of compound **5** in DMSO (300 µL) to the sterile Rico medium at the same final concentration. All flasks were incubated for an additional 5 days under the previously described conditions.

### 3.8. Extraction, Isolation, and Characterization of Biotransformation Products

The culture broth and mycelium were separated by vacuum filtration using a 200 µm pore size Nytal filter. The culture medium was saturated with NaCl and then extracted with EtOAc (×3). The organic layer was dried over anhydrous Na_2_SO_4_ and the solvent was removed using a rotary evaporated, yielding 106.0 mg of a crude extract. The resulting residue containing the biotransformation products was purified by column chromatography on silica gel, using an increasing gradient of EtOAc in *n*-hexane as mobile phase. Fractions collected were further purified by semipreparative and/or analytical HPLC to afford compounds **5** (21.6 mg), **6** (14.1 mg, 29% conversion), and **7** (22.0 mg, 45% conversion).

(2S,3S,4R,5R,6R,9S,10R,11S,12S,13S,15R)*-3,5,15-Triacetoxy-7,13-dideoxi-18-hydroxypremyrsinol* (**6**). Purified by semipreparative HPLC (*n*-hexane:EtOAc 30:70, t_R_ = 68 min, flow 3.0 mL/min). White amorphous powder;$\;{\left[\alpha \right]}_{D\;}^{21}$ = −16.0 (c 2.2, MeOH); ^1^H and ^13^C NMR data, see Table 1 and Figures S12 and S13; gHMBC (selected correlations) H-3 → C-1, C-15, OCO-3; H-4 → C-5, C-6, C-14; H-5 → C-3, C-4, C-6, C-7, C-15, C-17, OCO-5; H-9 → C-18; H-11 → C-8, C-9, C-10, C-13, C-18; H-13 → C-11, C-12, C-14, C-15, C-20; H_3_-16 → C-1, C-2, C-3; H_2_-17 → C-5, C-6, C-7, C-12; H_2_-18 → C-9, C-10, C-19; H_3_-19 → C-9, C-10, C-18; H_3_-20 → C-12, C-13, C-14; OCOMe-3 → OCO-3; OCOMe-5 → C-5, OCO-5; OCOMe-15 → C-15, OCO-15 (Figure S16); IR (film) *υ*_max_ 3517, 2935, 1738, 1372, 1240, 1024 cm^−1^; UV (MeOH) *λ*_max_ (log *ɛ*) 295 (1.41) nm; ECD (MeOH) *λ* (Δ*ε*) 204 (0.47), 218 (−0.63), 291 (−0.15) nm (Figure S20); HRESIMS [M + Na]^+^ *m/z* calcd for C_26_H_38_O_9_Na, 517.2414; found 517.2401 (Figure S19).

(2S,3S,4R,5R,6R,8S,9S,11R,12S,13S,15R)*-3,5,15-Triacetoxy-7,13-dideoxi-8-hydroxypremyrsinol* (**7**). Purificated by semipreparative HPLC (*n*-hexane:EtOAc 30:70, t_R_ = 96 min, flow 3.0 mL/min). White amorphous powder;$\;{\left[\alpha \right]}_{D\;}^{21}$ = −33.5 (c 2.1, MeOH); ^1^H and ^13^C NMR data, see Table 1 and Figures S21 and S22; gHMBC (selected correlations) H-3 → C-1, C-15, OCO-3; H-4 → C-5, C-6, C-14; H-5 → C-3, C-4, C-6, C-12, C-15, C-17; H-8 → C-7, C-9, C-10; H-11 → C-8, C-9, C-10, C-13, C-18, C-19; H-13 → C-11, C-12, C-14, C-15, C-20; H_2_-17 → C-5, C-6, C-7, C-12; H_3_-19 → C-9, C-10, C-11, C-18; OCOMe-15 → C-15, OCO-15 (Figure S25); IR (film) *υ*_max_ 3512, 2943, 1738, 1372, 1240, 1028 cm^−1^; UV (MeOH) *λ*_max_ (log *ɛ*) 295 (1.41) nm; ECD (MeOH) *λ* (Δ*ε*) 202 (2.04), 219 (−0.67), 292 (−0.30) nm (Figure S29); HRESIMS [M + Na]^+^ *m/z* calcd for C_26_H_38_O_9_Na, 517.2414; found 517.2405 (Figure S28). 

## 4. Conclusions

An efficient catalytic synthetic approach has been development using epoxyboetirane A (**4**) as starting material to afford a new premyrsinane-type diterpenoid (**5**) via a diasteroselective transannular cyclization with Cp_2_Ti^III^Cl. This biomimetic cyclization could reinforce the Appendino’s proposal on the biogenetic origin of premyrsinanes from 6,17-epoxylathrane-type diterpenoids. Biotransformation of compound **5** by *M. circinelloides* NRRL3631 has allowed to obtain oxidized derivatives unattainable using chemical means. In particular, two derivatives (**6**–**7**) were obtained by regioselective hydroxylation of **5** at C-18 and C-8 positions. 

These derivatives offer a promising scaffold for their evaluation as potential neuroregenerative agents. Therefore, it would be interesting to investigate the capability of these premyrsinane-type diterpenoids to induce the release of transforming growth factor alpha (TFGα) and neuregulin 1 (NRG1) in NSC cultures. This assessment will serve as an indirect means of evaluating the abovementioned compounds’ potential to promote neurogenesis.

## Figures and Tables

**Table 1:** ^1^H (400 MHz) and ^13^C NMR (100 MHz) spectroscopic data for compounds **5**–**7** in CDCl_3_.

Position	5		6		7	
	*δ*_H_, Mult (*J* in Hz)	*δ*_C_, Type	*δ*_H_, Mult (*J* in Hz)	*δ*_C,_ Type	*δ*_H_*,* Mult (*J* in Hz)	*δ*_C,_ Type
1*α*	3.36, dd (15.1, 9.1)	42.3, CH_2_	3.35, dd (15.1, 8.9)	42.3, CH_2_	3.35, dd (15.1, 8.9)	42.4, CH_2_
1*β*	1.54, dd (15.1, 11.1)		1.55, dd (15.1, 11.2)		1.55, dd (15.1, 11.2)	
2	2.14–2.06, m	37.0, CH	2.11–2.06, m	37.0, CH	2.13–2.06, m	37.3, CH
3	5.34, t (3.6)	78.5, CH	5.34, t (3.5)	78.4, CH	5.35, t (3.5)	78.4, CH
4	2.65, dd (11.4, 3.6)	51.9, CH	2.66, dd (11.4, 3.5)	51.9, CH	2.63, dd (11.4, 3.5)	51.8, CH
5	5.41, d (11.4)	74.7, CH	5.42, d (11.4)	74.7, CH	5.50, d (11.4)	75.1, CH
6	-	44.1, C	-	44.1, C	-	48.0, C
7*α*	1.40, dt (14.1, 6.4)	30.6, CH_2_	1.45, dd (13.8, 5.9)	30.5, CH_2_	1.92, dd (13.8, 7.6)	39.2, CH_2_
7*β*	1.15–1.09, m		1.12–1.05, m		1.02, dd (13.8, 11.6)	
8*α*	1.76–1.63, m	15.8, CH_2_	1.80–1.70, m	15.4, CH_2_	4.37, dt (11.6, 7.6)	64.9, CH
8*β*	1.33–1.22, m		1.40–1.33, m		-	
OH-8	-	-	-	-	1.31, d (5.2)	-
9	0.70, ddd (9.4, 7.9, 4.5)	21.7, ^a^ CH	0.85, ddd (9.6, 7.5, 3.9)	18.2, CH	1.06, dd (9.4, 7.6)	26.3, CH
10	-	18.0, C	-	24.3, C	-	20.7, C
11	0.11, dd (9.4, 6.7)	27.1, CH	0.34, dd (9.6, 6.2)	24.0, CH	0.40, dd (9.4, 4.8)	32.0, CH
12	1.76–1.63, m	38.0, CH	1.75, dd (11.4, 6.2)	37.5, CH	1.88, dd (11.7, 4.8)	38.9, CH
13	2.72–2.61, m	50.6, CH	2.73, dq (11.4, 7.5)	51.0, CH	2.58, dt (11.7, 7.4)	50.7, CH
14	-	209.0, C	-	208.7, C	-	208.3, C
15	-	91.8, C	-	91.7, C	-	91.4, C
16	0.87, d (6.7)	14.1, CH_3_	0.87, d (6.7)	14.0, CH_3_	0.88, d (6.7)	13.9, CH_3_
17a	3.84, d (11.3)	63.2, CH_2_	3.87, dd (11.3, 2.5)	62.8, CH_2_	3.86, dd (11.7, 2.5)	61.8, CH_2_
17b	3.68, d (11.3)		3.71, dd (11.3, 6.9)		3.67, dd (11.7, 8.2)	
OH-17	-	-	1.67, dd (6.9, 2.5)	-	1.68, dd (8.2, 2.5)	-
18a	1.03, s	28.2, CH_3_	3.41, d (10.4)	72.8, CH_2_	1.12, s	29.5, CH_3_
18b			3.30, d (10.4)			
19	0.99, s	16.1, CH_3_	1.11, s	11.7, CH_3_	1.24, s	17.4, CH_3_
20	1.10, d (7.5)	19.7, CH_3_	1.13, d (7.5)	19.7, CH_3_	1.12, d (7.4)	20.3, CH_3_
OCO-3	-	171.0, C	-	170.0, C	-	171.0, ^e^ C
OCOMe-3	2.04, s	21.0, ^b^ CH_3_	2.03, s	21.0, ^c^ CH_3_	2.04, s	21.2, ^d^ CH_3_
OCO-5	-	170.0, C	-	171.0, C	-	170.0, ^e^ C
OCOMe-5	2.03, s	21.2, ^b^ CH_3_	2.04, s	21.2, ^c^ CH_3_	2.04, s	21.0, ^d^ CH_3_
OCO-15	-	169.7, C	-	169.7, C	-	170.0, C
OCOMe-15	2.12, s	21.6, ^a^ CH_3_	2.14, s	21.6, CH_3_	2.15, s	21.6, CH_3_

^a–e^ Interchangeable signals.

## Data Availability

The data presented in this study are available in Supplementary Materials.

## Supplementary Materials

The following supporting information can be downloaded at: https://www.mdpi.com/article/10.3390/plants13060842/s1, Table S1: ^1^H and ^13^C NMR spectroscopic data of **4**; Figure S1: Selected NOESY correlations for **4**; Figure S2: ^1^H NMR spectra of synthetic and isolated compound **4**; Figures S3–S29: 1D and 2D NMR, ECD and HRESIMS spectra of compounds **5**–**7**.

## References

[1] Zhan Z., Li S., Chu W., Yin S. (2022). *Euphorbia* Diterpenoids: Isolation, Structure, Bioactivity, Biosynthesis, and Synthesis (2013–2021). Nat. Prod. Rep..

[2] Durán-Peña M.J., Botubol Ares J.M., Collado I.G., Hernández-Galán R. (2014). Biologically Active Diterpenes Containing a *gem*-Dimethylcyclopropane Subunit: An Intriguing Source of PKC Modulators. Nat. Prod. Rep..

[3] Vasas A., Hohmann J. (2014). *Euphorbia* Diterpenes: Isolation, Structure, Biological Activity, and Synthesis (2008–2012). Chem. Rev..

[4] Benjamaa R., Moujanni A., Kaushik N., Choi E.H., Essamadi A.K., Kaushik N.K. (2022). *Euphorbia* Species Latex: A Comprehensive Review on Phytochemistry and Biological Activities. Front. Plant Sci..

[5] Kemboi D., Siwe-Noundou X., Krause R.W.M., Langat M.K., Tembu V.J. (2021). *Euphorbia* Diterpenes: An Update of Isolation, Structure, Pharmacological Activities and Structure–Activity Relationship. Molecules.

[6] Fattahian M., Ghanadian M., Ali Z., Khan I.A. (2020). Jatrophane and Rearranged Jatrophane-Type Diterpenes: Biogenesis, Structure, Isolation, Biological Activity and SARs (1984–2019). Phytochem. Rev..

[7] Wang H.-B., Wang X.-Y., Liu L.-P., Qin G.-W., Kang T.-G. (2015). Tigliane Diterpenoids from the *Euphorbiaceae* and *Thymelaeaceae* Families. Chem. Rev..

[8] Alves A.L.V., da Silva L.S., Faleiros C.A., Silva V.A.O., Reis R.M. (2022). The Role of Ingenane Diterpenes in Cancer Therapy: From Bioactive Secondary Compounds to Small Molecules. Nat. Prod. Commun..

[9] Vela F., Ezzanad A., Hunter A.C., Macías-Sánchez A.J., Hernández-Galán R. (2022). Pharmacological Potential of Lathyrane-Type Diterpenoids from Phytochemical Sources. Pharmaceuticals.

[10] Mendes E., Ramalhete C., Duarte N. (2024). Myrsinane-Type Diterpenes: A Comprehensive Review on Structural Diversity, Chemistry and Biological Activities. Int. J. Mol. Sci..

[11] Hegazy M.-E., Hamed A., Ibrahim M., Talat Z., Reda E., Abdel-Azim N., Hammouda F., Nakamura S., Matsuda H., Haggag E. (2018). Euphosantianane A–D: Antiproliferative Premyrsinane Diterpenoids from the Endemic Egyptian Plant *Euphorbia sanctae-catharinae*. Molecules.

[12] Jeske F., Jakupovic J., Berendsohn W. (1995). Diterpenes from *Euphorbia seguieriana*. Phytochemistry.

[13] Shi Q.-W., Su X.-H., Kiyota H. (2008). Chemical and Pharmacological Research of the Plants in Genus *Euphorbia*. Chem. Rev..

[14] Appendino G., Cravotto G., Jarevång T., Sterner O. (2000). Epoxidation Studies on Lathyra-6(17),12-Dienes—Revised Structure of the Euphorbia Factor L_1_. Eur. J. Org. Chem..

[15] Xiao Y., Wang N., Wan L.X., Zhou X.L., Li X., Gao F. (2021). Iron-Catalyzed Skeletal Conversion of Lathyrane to Premyrsinane *Euphorbia* Diterpenes and Their Cytotoxic Activities. J. Nat. Prod..

[16] Xiao Y., Zhang Y., Ji W.-S., Jia X.-N., Shan L.-H., Li X., Liu Y.-J., Jiang T., Gao F. (2023). Discovery of Myrsinane-Type *Euphorbia* Diterpene Derivatives through a Skeleton Conversion Strategy from Lathyrane Diterpene for the Treatment of Alzheimer’s Disease. Bioorg. Chem..

[17] Gao J., Chen Q.-B., Liu Y.-Q., Xin X.-L., Yili A., Aisa H.A. (2016). Diterpenoid Constituents of *Euphorbia macrorrhiza*. Phytochemistry.

[18] Yazdiniapour Z., Sohrabi M.H., Motinia N., Zolfaghari B., Mehdifar P., Ghanadian M., Lanzotti V. (2023). Diterpenoids from *Euphorbia gedrosiaca* as Potential Anti-Proliferative Agents against Breast Cancer Cells. Metabolites.

[19] Zolfaghari B., Farahani A., Jannesari A., Aghaei M., Ghanadian M. (2022). New Cytotoxic Premyrsinane-Type Diterpenes from *Euphorbia aleppica* against Breast Cancer Cells. Iran. J. Pharm. Res..

[20] Vasas A., Sulyok E., Martins A., Rédei D., Forgo P., Kele Z., Zupkó I., Molnár J., Pinke G., Hohmann J. (2012). Cyclomyrsinane and Premyrsinane Diterpenes from *Euphorbia falcata* Modulate Resistance of Cancer Cells to Doxorubicin. Tetrahedron.

[21] Xu J., Jin D., Guo Y., Xie C., Ma Y., Yamakuni T., Ohizumi Y. (2012). New Myrsinol Diterpenes from *Euphorbia prolifera* and Their Inhibitory Activities on LPS-Induced NO Production. Bioorg. Med. Chem. Lett..

[22] Flores-Giubi E., Geribaldi-Doldán N., Murillo-Carretero M., Castro C., Durán-Patrón R., Macías-Sánchez A.J., Hernández-Galán R. (2019). Lathyrane, Premyrsinane, and Related Diterpenes from *Euphorbia boetica*: Effect on In Vitro Neural Progenitor Cell Proliferation. J. Nat. Prod..

[23] Botubol-Ares J.M., Durán-Peña M.J., Hanson J.R., Hernández-Galán R., Collado I.G. (2018). Cp_2_Ti(III)Cl and Analogues as Sustainable Templates in Organic Synthesis. Synthesis.

[24] Streuff J. (2023). Reductive Umpolung and Defunctionalization Reactions through Higher-Order Titanium(III) Catalysis. Synlett.

[25] Rosales A., Rodríguez-García I., Muñoz-Bascõn J., Roldan-Molina E., Padial N.M., Morales L.P., García-Ocaña M., Oltra J.E. (2015). The Nugent Reagent: A Formidable Tool in Contemporary Radical and Organometallic Chemistry. Eur. J. Org. Chem..

[26] Morcillo S.P., Miguel D., Campaña A.G., Álvarez De Cienfuegos L., Justicia J., Cuerva J.M. (2014). Recent Applications of Cp_2_TiCl in Natural Product Synthesis. Org. Chem. Front..

[27] Martínez A.R., Morales L.P., Ojeda E.D., Rodríguez M.C., Rodríguez-García I. (2021). The Proven Versatility of Cp_2_TiCl. J. Org. Chem..

[28] Green M.L.H., Lucas C.R. (1972). Some d^l^ Bis-π-cyclopentadienyl Titanium Complexes with Nitrogen or Phosphorus Ligands. J. Chem. Soc. Dalton Trans..

[29] Gansäuer A., Bluhm H., Pierobon M. (1998). Emergence of a Novel Catalytic Radical Reaction: Titanocene-Catalyzed Reductive Opening of Epoxides. J. Am. Chem. Soc..

[30] Nugent W.A., RajanBabu T.V. (1988). Transition-Metal-Centered Radicals in Organic Synthesis. Titanium(III)-Induced Cyclization of Epoxy Olefins. J. Am. Chem. Soc..

[31] Barrero A.F., Quílez del Moral J.F., Sánchez E.M., Arteaga J.F. (2006). Titanocene-Mediated Radical Cyclization: An Emergent Method Towards the Synthesis of Natural Products. Eur. J. Org. Chem..

[32] Valentová K. (2023). Biotransformation of Natural Products and Phytochemicals: Metabolites, Their Preparation, and Properties. Int. J. Mol. Sci..

[33] Pinedo-Rivilla C., Moraga J., Pérez-Sasián G., Peña-Hernández A., Collado I.G., Aleu J. (2020). Biocatalytic Preparation of Chloroindanol Derivatives. Antifungal Activity and Detoxification by the Phytopathogenic Fungus *Botrytis cinerea*. Plants.

[34] Rico-Martínez M., Medina F.G., Marrero J.G., Osegueda-Robles S. (2014). Biotransformation of Diterpenes. RSC Adv..

[35] De Sousa I.P., Sousa Teixeira M.V., Jacometti Cardoso Furtado N.A. (2018). An Overview of Biotransformation and Toxicity of Diterpenes. Molecules.

[36] Wu Y., Yin C., Cao Y., Liu X., Yu J., Zhang J., Yu B., Cheng Z. (2016). Regioselective Hydroxylation of Lathyrane Diterpenoids Biocatalyzed by Microorganisms and Its Application in Integrated Synthesis. J. Mol. Catal. B-Enzym..

[37] Liu X., Cheng Z. (2023). Microbial and Chemical Transformations of Euphorbia Factor L_1_ and the P-Glycoprotein Inhibitory Activity in Zebrafishes. Nat. Prod. Res..

[38] Xiao S., Xu X., Wei X., Xin J., Li S., Lv Y., Chen W., Yuan W., Xie B., Zu X. (2022). Comprehensive Metabolic Profiling of Euphorbiasteroid in Rats by Integrating UPLC-Q/TOF-MS and NMR as Well as Microbial Biotransformation. Metabolites.

[39] Escobar-Montaño F., González-Rodríguez V.E., Macías-Sánchez A.J., Botubol-Ares J.M., Durán-Patrón R., Hernández-Galán R. (2024). Enhancing Structural Diversity of Lathyrane Derivatives through Biotransformation by the Marine-Derived Actinomycete *Streptomyces puniceus* BC-5GB.11. Int. J. Mol. Sci..

[40] Vieira C., Duarte N., Reis M.A., Spengler G., Madureira A.M., Molnár J., Ferreira M.-J.U. (2014). Improving the MDR Reversal Activity of 6,17-Epoxylathyrane Diterpenes. Bioorg. Med. Chem..

[41] Engel D.W. (1972). The Determination of Absolute Configuration and Δ*f*” Values for Light-Atom Structures. Acta Crystallog. B.

[42] Engel D.W., Zechmeister K., Hoppe W. (1972). Experience in the Determination of Absolute Configuration of Light Atom Compounds by X-Ray Anomalous Scattering. Tetrahedron Lett..

[43] Lu J., Li G., Huang J., Zhang C., Zhang L., Zhang K., Li P., Lin R., Wang J. (2014). Lathyrane-Type Diterpenoids from the Seeds of *Euphorbia lathyris*. Phytochemistry.

[44] Jiao W., Mao Z., Dong W., Deng M., Lu R.-H. (2008). Euphorbia Factor L_8_: A Diterpenoid from the Seeds of It *Euphorbia lathyris*. Acta Crystallogr. E.

[45] Justicia J., Rosales A., Buñuel E., Oller-López J.L., Valdivia M., Haïdour A., Oltra J.E., Barrero A.F., Cárdenas D.J., Cuerva J.M. (2004). Titanocene-Catalyzed Cascade Cyclization of Epoxypolyprenes: Straightforward Synthesis of Terpenoids by Free-Radical Chemistry. Chem.–Eur. J..

[46] Gansaeuer A., Pierobon M., Bluhm H. (2004). Titanocene Catalysed 5-Exo Cyclisations of Unsaturated Epoxides—Reagent Control in Radical Chemistry. Synthesis.

[47] Estévez R.E., Justicia J., Bazdi B., Fuentes N., Paradas M., Choquesillo-Lazarte D., García-Ruiz J.M., Robles R., Gansäuer A., Cuerva J.M. (2009). Ti-Catalyzed Barbier-Type Allylations and Related Reactions. Chem.–Eur. J..

[48] Escobar-Montaño F., Macías-Sánchez A.J., Botubol-Ares J.M., Durán-Patrón R., Hernández-Galán R. (2024).

