# Supplementary material for: A Biomimetic Approach to Premyrsinane-Type Diterpenoids: Exploring Microbial Transformation to Enhance Their Chemical Diversity
Source: Plants (Basel). 2024 Mar 14;13(6):842. doi: 10.3390/plants13060842 (PMC10975351; doi:10.3390/plants13060842)
Supplement: Supplementary file 1 [file plants-13-00842-s001.zip › plants-2908491-supplementary.pdf]

A Biomimetic Approach to Premyrsinane-Type Diterpenoids: Exploring Microbial Transformation to Enhance Their Chemical Diversity

Felipe Escobar-Montaño [1], Antonio J. Macías-Sánchez [1,2], José M. Botubol-Ares [1,3,*], Rosa Durán-Patrón [1,2,*] and Rosario Hernández-Galán [1,2]

1 Departamento de Química Orgánica, Facultad de Ciencias, Universidad de Cádiz, Puerto Real, 11510 Cádiz, Spain

2 Instituto Universitario de Investigación en Biomoléculas, Universidad de Cádiz, Puerto Real, 11510 Cádiz, Spain

3 Instituto Universitario de Investigación Vitivinícola y Agroalimentaria, Universidad de Cádiz, Puerto Real, 11510 Cádiz, Spain

* Correspondence: josemanuel.botubol@uca.es (J.M.B.-A.); rosa.duran@uca.es (R.D.-P.)

Table of contents

Table S1. ^{1}H (400 MHz) and ^{13}C NMR (100 MHz) spectroscopic data for **4** in $CDCl_3$ S3

Figure S1. Selected 2D NOESY correlations for compound **4** S4

Figure S2. ^{1}H NMR spectra (400 MHz) of synthetic and isolated compound **4** in $CDCl_3$ S5

Figure S3. ^{1}H NMR spectrum (400 MHz) of compound **5** in $CDCl_3$ S6

Figure S4. ^{13}C NMR spectrum (100 MHz) of compound **5** in $CDCl_3$ S7

Figure S5. gCOSY spectrum of compound **5** S8

Figure S6. gHSQC spectrum of compound **5** S9

Figure S7. gHMBC spectrum of compound **5** S10

Figure S8. 2D NOESY spectrum of compound **5** S11

Figures S9a-e. 1D NOESY spectra of compound **5** S12-16

Figure S10. HRMS of compound **5** S17

Figure S11. ECD spectrum of compound **5** S18

Figure S12. ^{1}H NMR spectrum (400 MHz) of compound **6** in $CDCl_3$ S19

Figure S13. ^{13}C NMR spectrum (100 MHz) of compound **6** in $CDCl_3$ S20

Figure S14. gCOSY spectrum of compound **6** S21

Figure S15. gHSQC spectrum of compound **6** S22

Figure S16. gHMBC spectrum of compound **6** S23

Figure S17. 2D NOESY spectrum of compound **6** S24

Figures S18a-b. 1D NOESY spectra of compound **6** S25-26

Figure S19. HRMS of compound **6** S27

Figure S20. ECD spectrum of compound **6** S28

Figure S21. ^{1}H NMR spectrum (400 MHz) of compound **7** in $CDCl_3$ S29

Figure S22. ^{13}C NMR spectrum (100 MHz) of compound **7** in $CDCl_3$ S30

Figure S23. gCOSY spectrum of compound **7** S31

Figure S24. gHSQC spectrum of compound **7** S32

Figure S25. gHMBC spectrum of compound **7** S33

Figure S26. 2D NOESY spectrum of compound **7** S34

Figures S27a-c. 1D NOESY spectra of compound **7** S35-37

Figure S28. HRMS of compound **7** S38

Figure S29. ECD spectrum of compound **7** S39

Table S1. ^{1}H (400 MHz) and ^{13}C NMR (100 MHz) spectroscopic data for **4** in $CDCl_3$.

Position	δ_H, mult (J in Hz)	δ_C, type
1α	3.40, dd (14.2, 8.0)	47.9, CH_2
1β	1.52, dd (14.2, 11.2)	
2α	2.13,[a] m	37.6, CH
3α	5.45, t (3.4)	80.3, CH
4α	1.89, dd (9.4, 3.4)	50.0, CH
5β	6.20, d (9.4)	65.2, CH
6	-	58.9, C
7α	0.92, m	33.5, CH_2
7β	2.11,[a] m	
8α	2.07,[a] m	20.0, CH_2
8β	1.71, dt (16.2, 12.0)	
9α	1.07, m	34.8, CH
10	-	25.6, C
11α	1.48, dd (11.4, 8.0)	29.0, CH
12	6.58, dd (11.4, 1.2)	143.7, CH
13	-	136.0, C
14	-	196.9, C
15	-	91.8, C
16	0.86, d (6.8)	14.0, CH_3
17a	2.48, d (3.2)	55.4, CH_2
17b	2.31, dd (3.2, 2.4)	
18	1.20,[b] s	28.9, CH_3
19	1.20,[b] s	16.7, CH_3
20	1.85, d (1.2)	12.4, CH_3
O<u>C</u>O-3	-	170.3,[d] C
OCO<u>Me</u>-3	2.00,[c] s	20.8,[e] CH_3
O<u>C</u>O-5	-	170.6, C
OCO<u>Me</u>-5	2.06, s	21.0, CH_3
O<u>C</u>O-15	-	169.6,[d] C
OCO<u>Me</u>-15	2.09,[c] s	21.8,[e] CH_3

[a-b] overlapped signals;[c-e] interchangeable signals

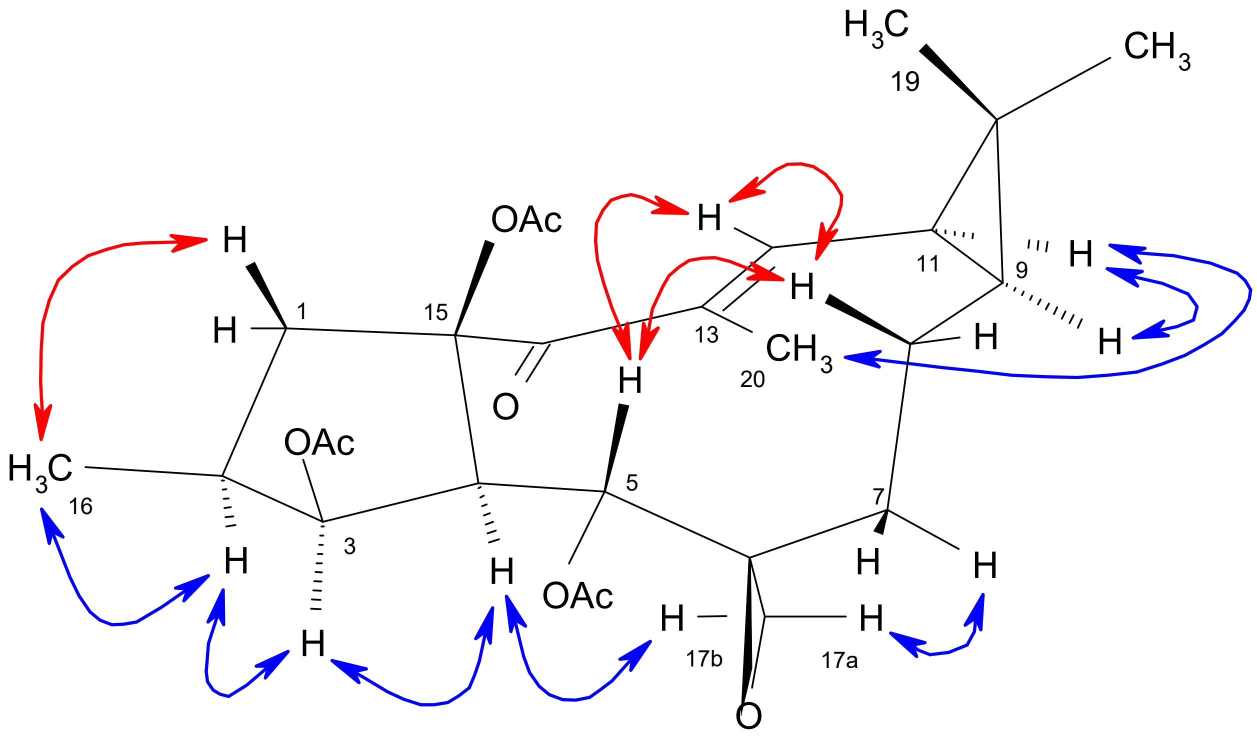

Figure S1. Selected 2D NOESY correlations for compound **4**. β-Face correlations in red. α-Face correlations in blue. Correlations involving nuclei in α-face and β-face in black.

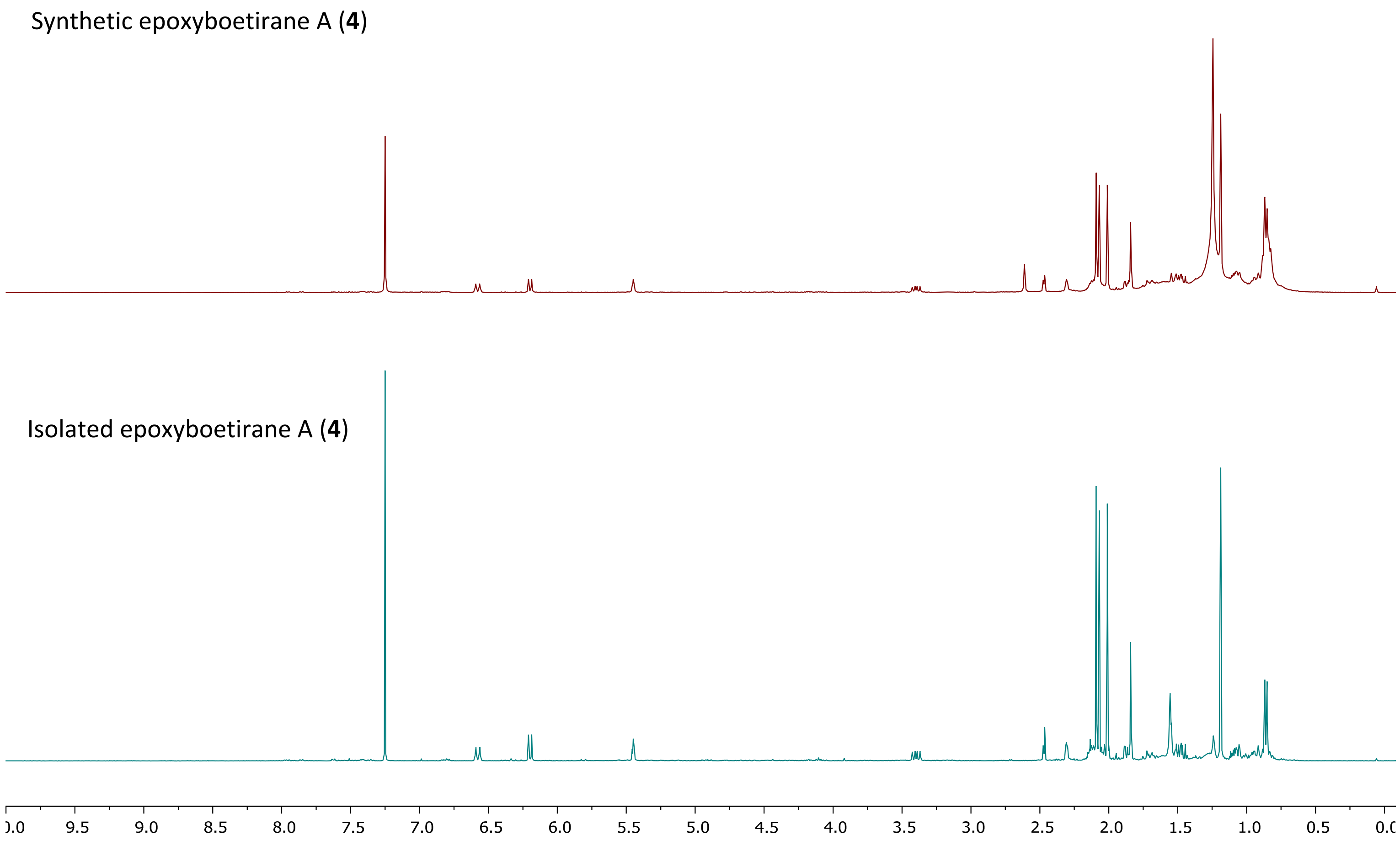

Figure S2. ^{1}H NMR spectra (400 MHz) of synthetic and natural compound **4** in $CDCl_3$.

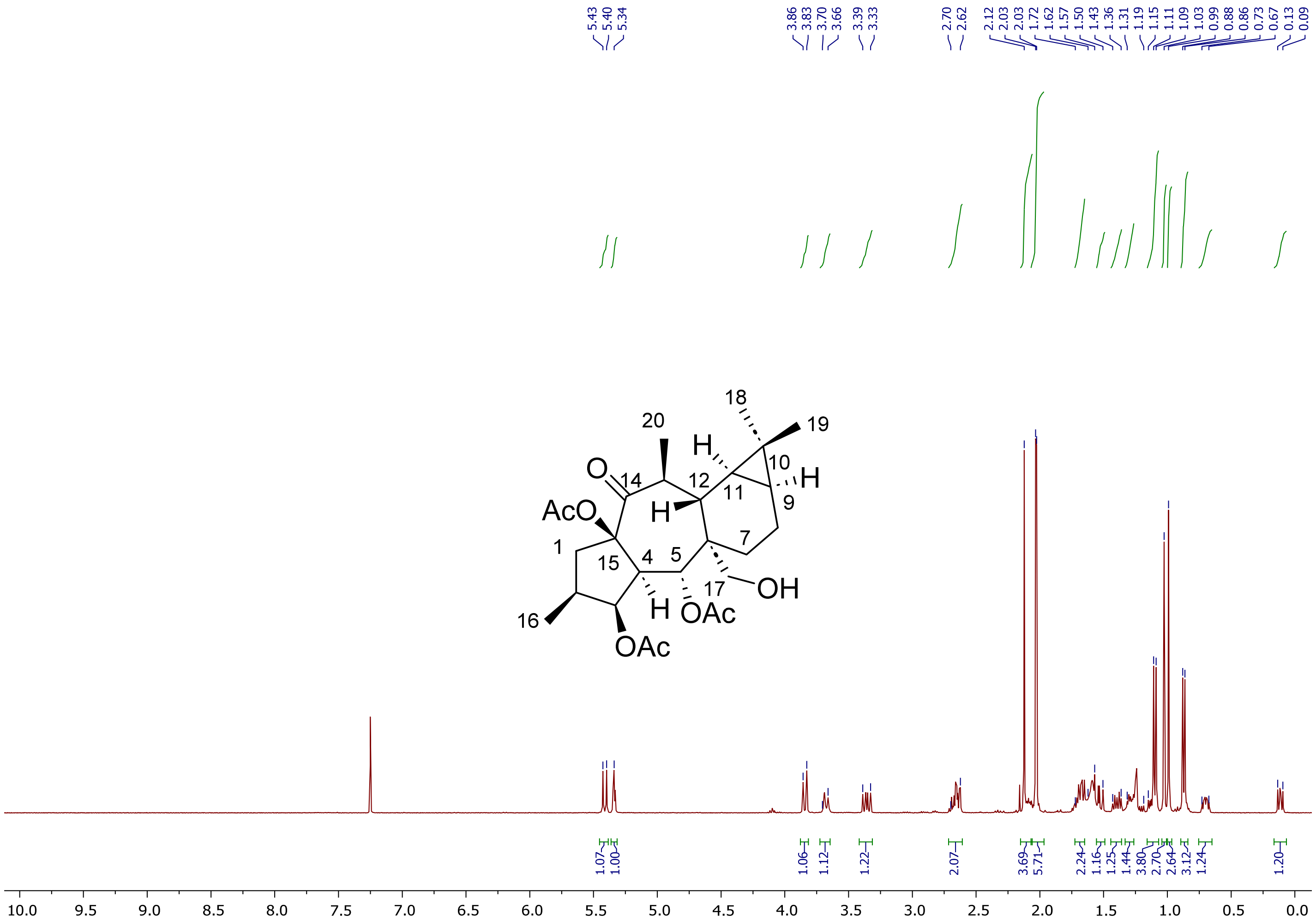

Figure S3. ^{1}H NMR spectrum (400 MHz) of compound **5** in $CDCl_3$.

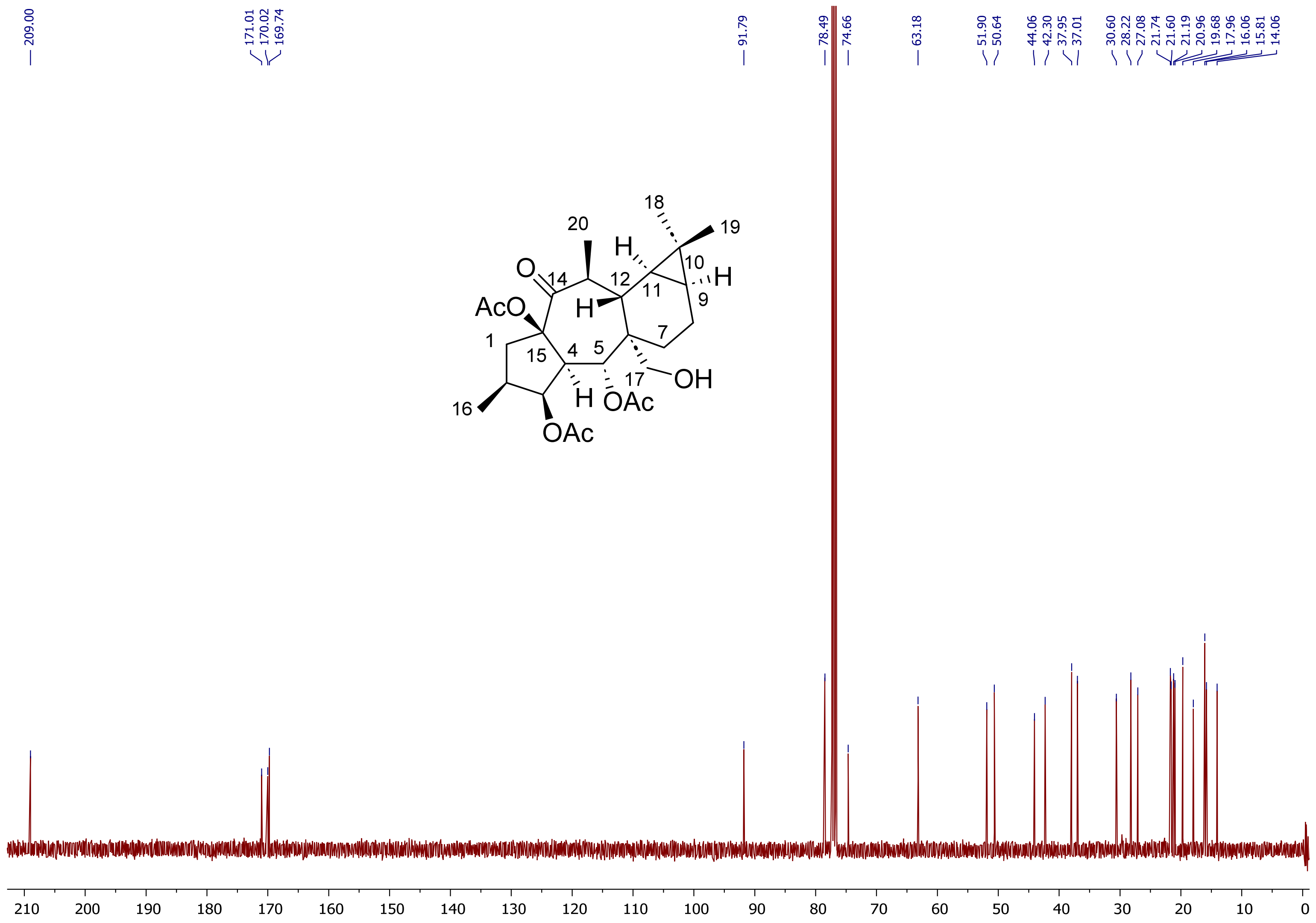

Figure S4. ^{13}C NMR spectrum (100 MHz) of compound **5** in $CDCl_3$.

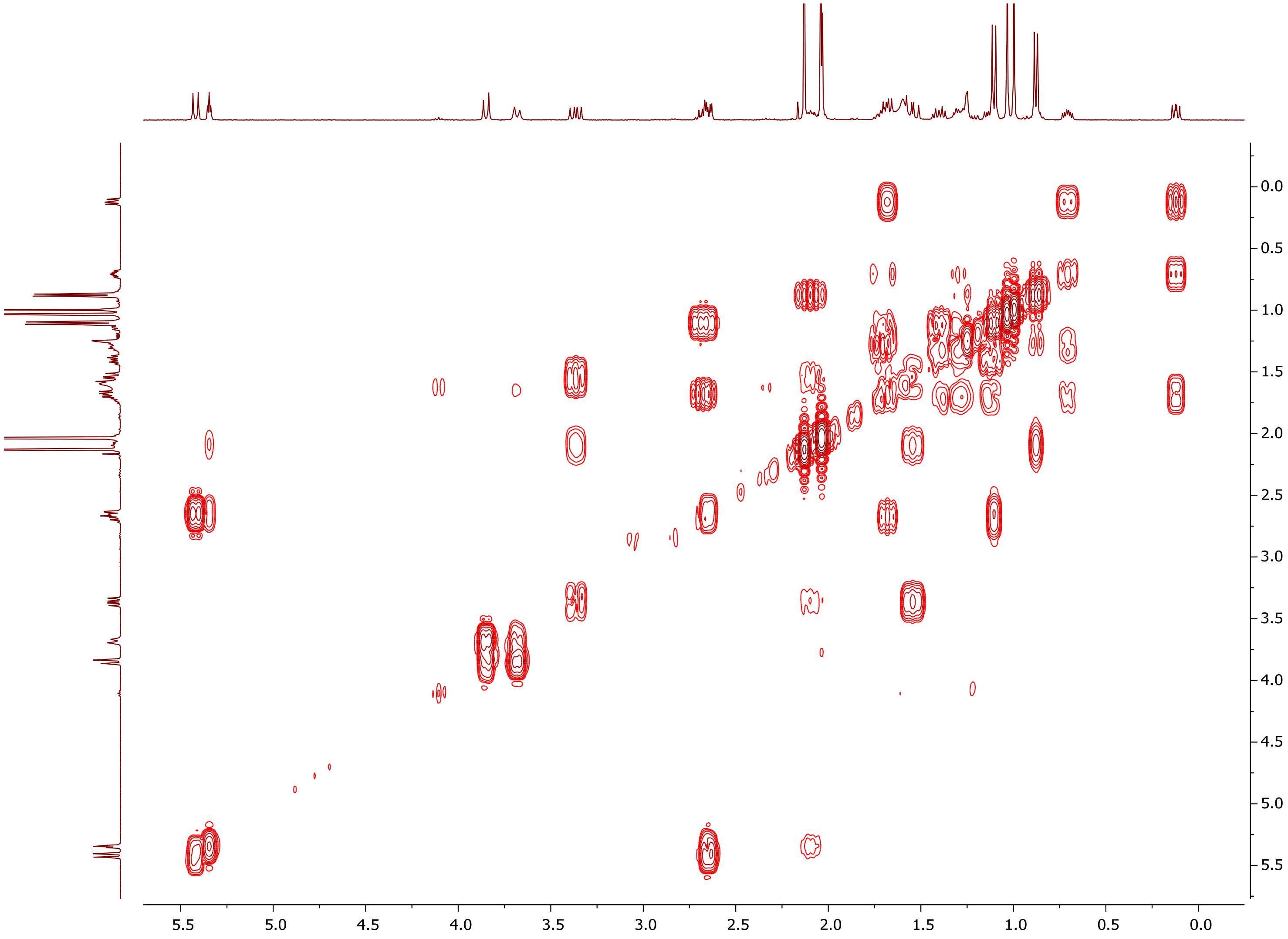

Figure S5. gCOSY spectrum of compound **5**.

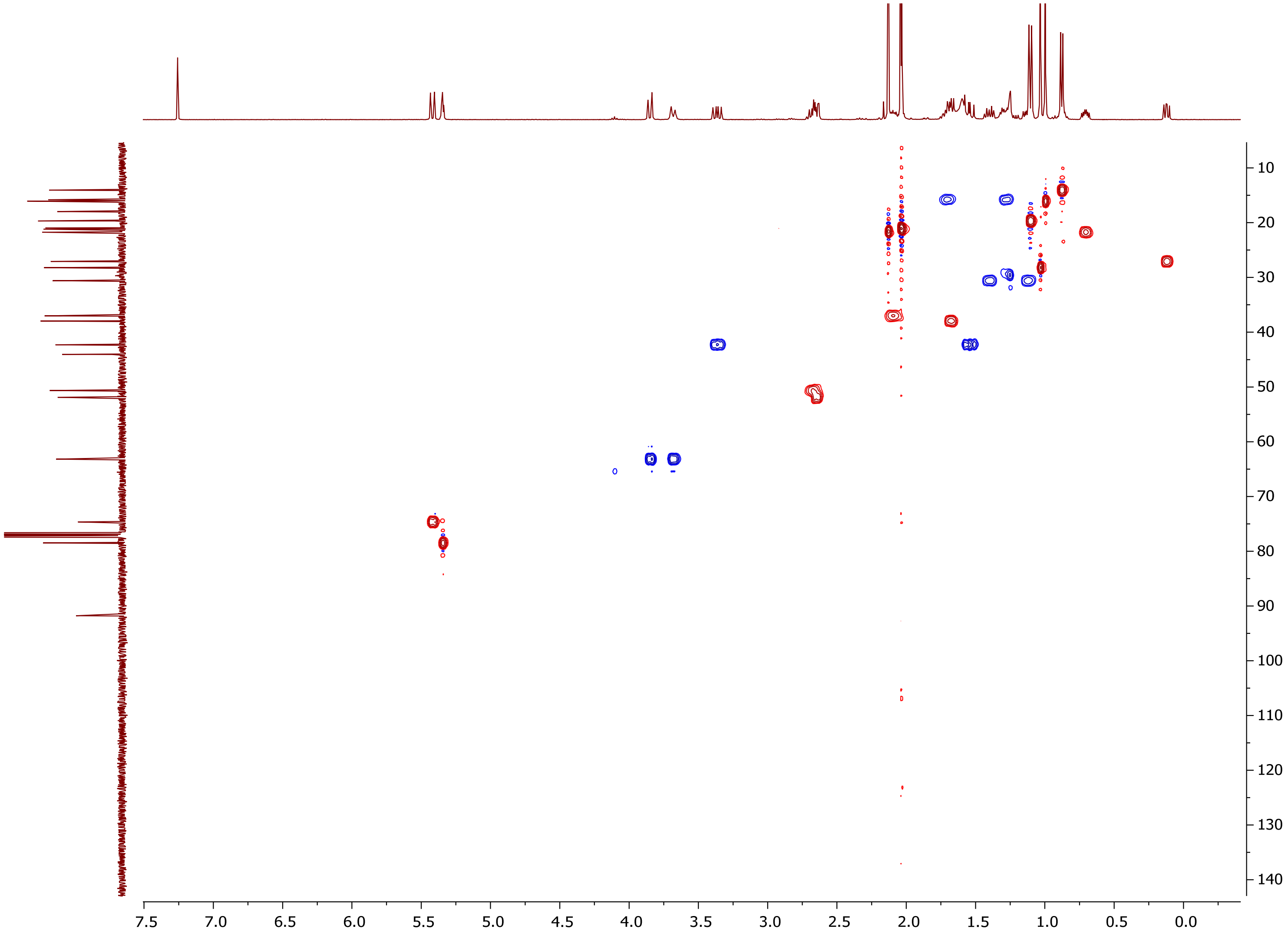

Figure S6. gHSQC spectrum of compound **5**.

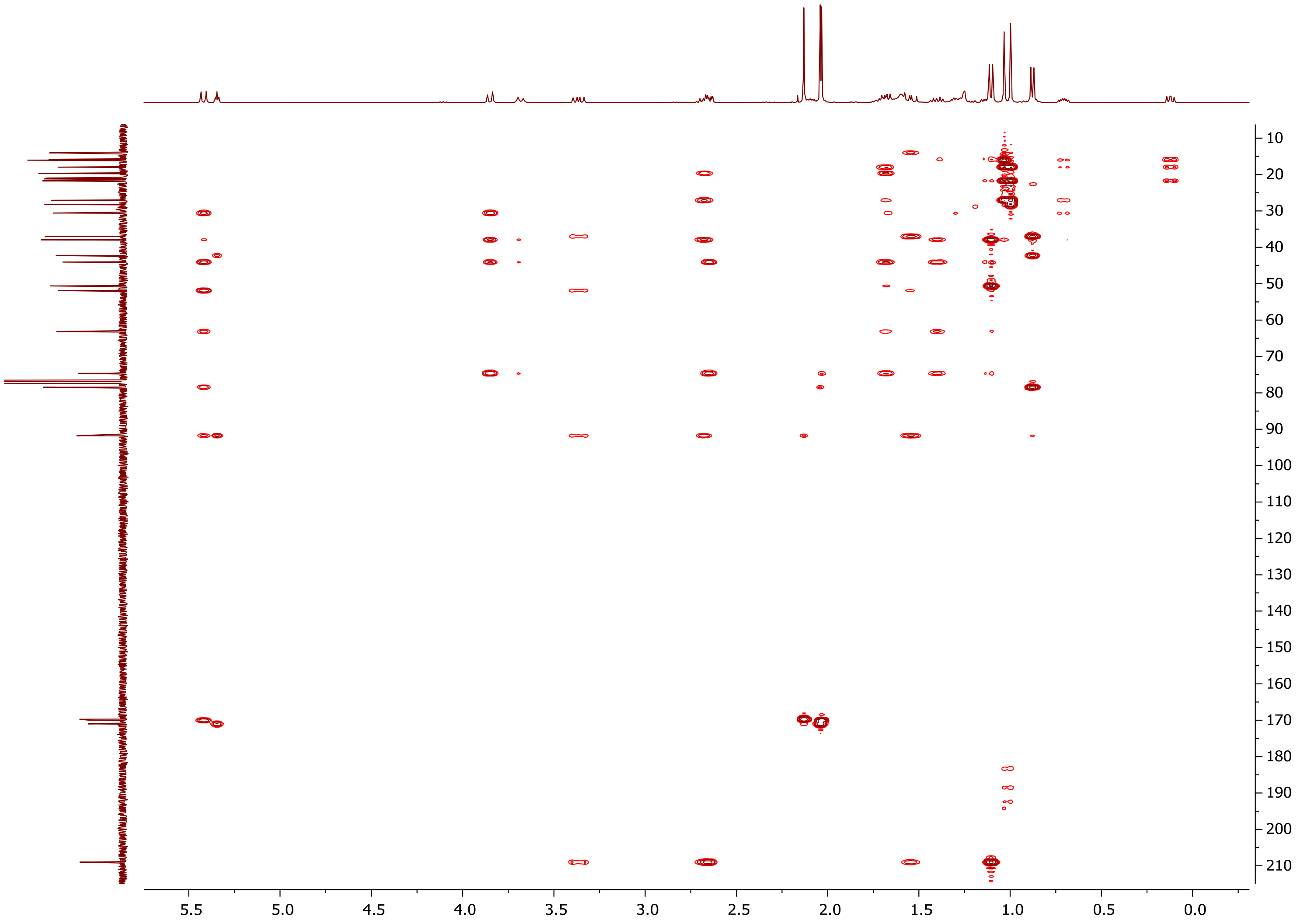

Figure S7. gHMBC spectrum of compound **5**.

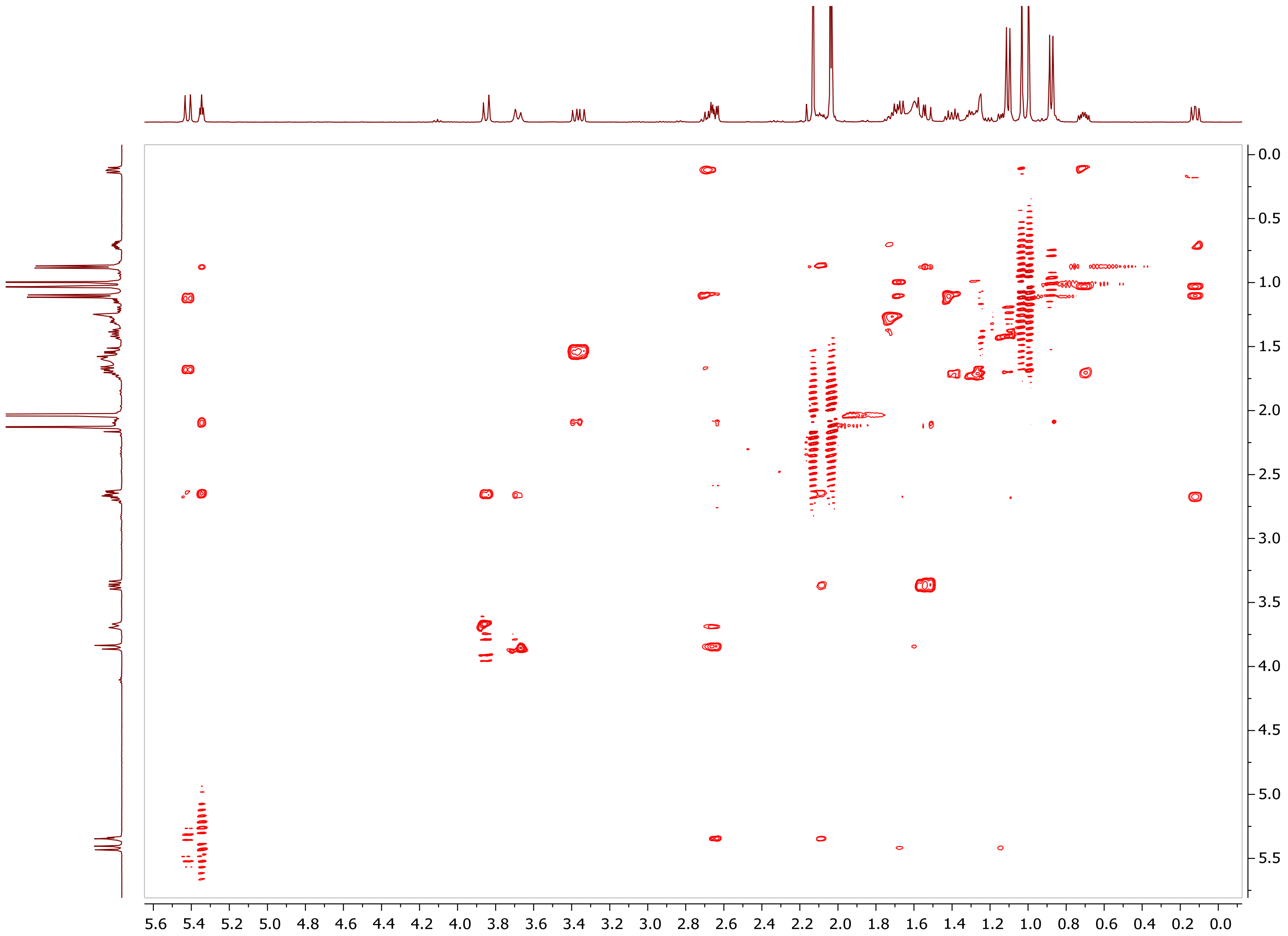

Figure S8. 2D NOESY spectrum of compound **5**.

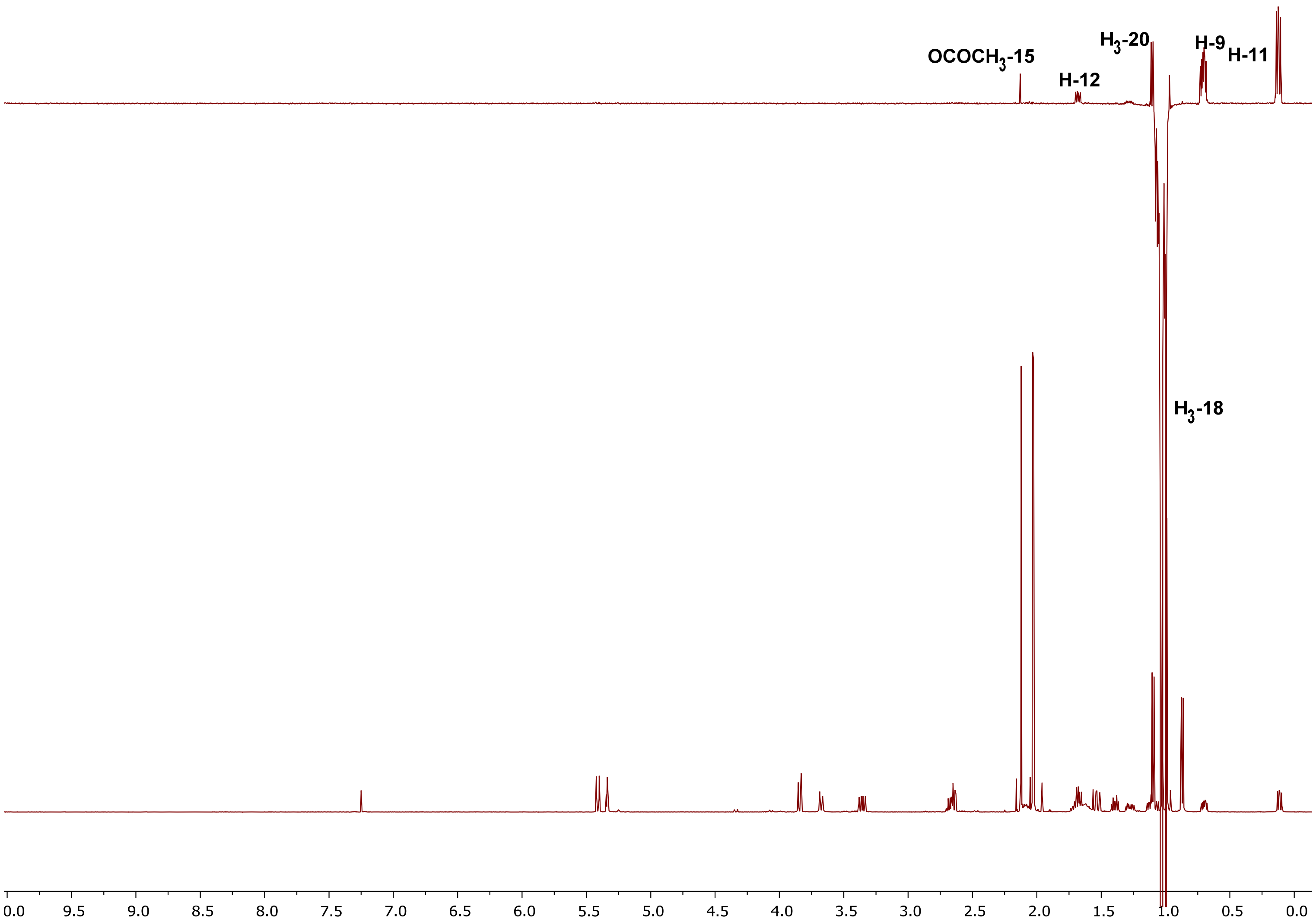

Figure S9a. 1D NOESY spectrum of compound **5**.

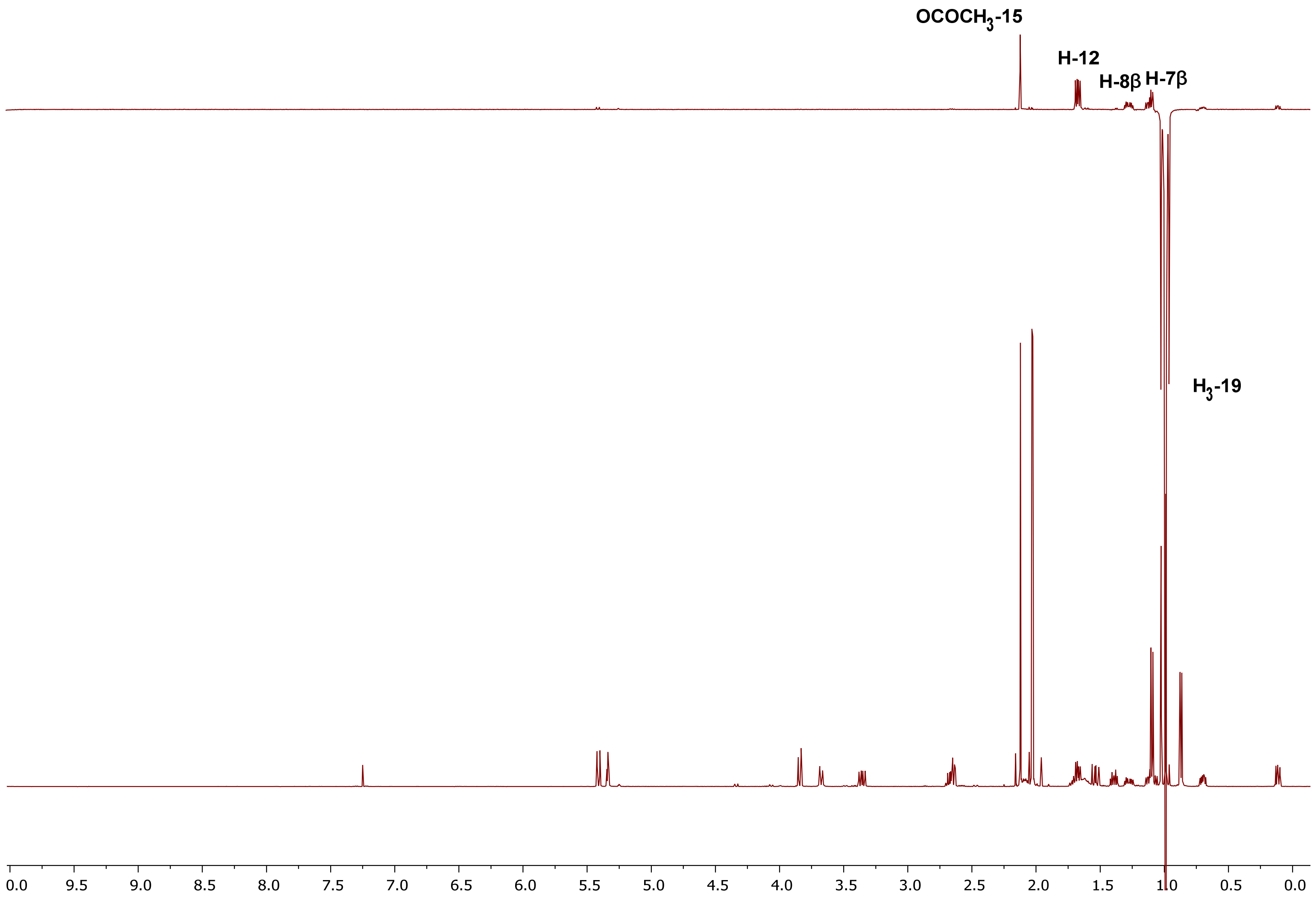

Figure S9b. 1D NOESY spectrum of compound **5**.

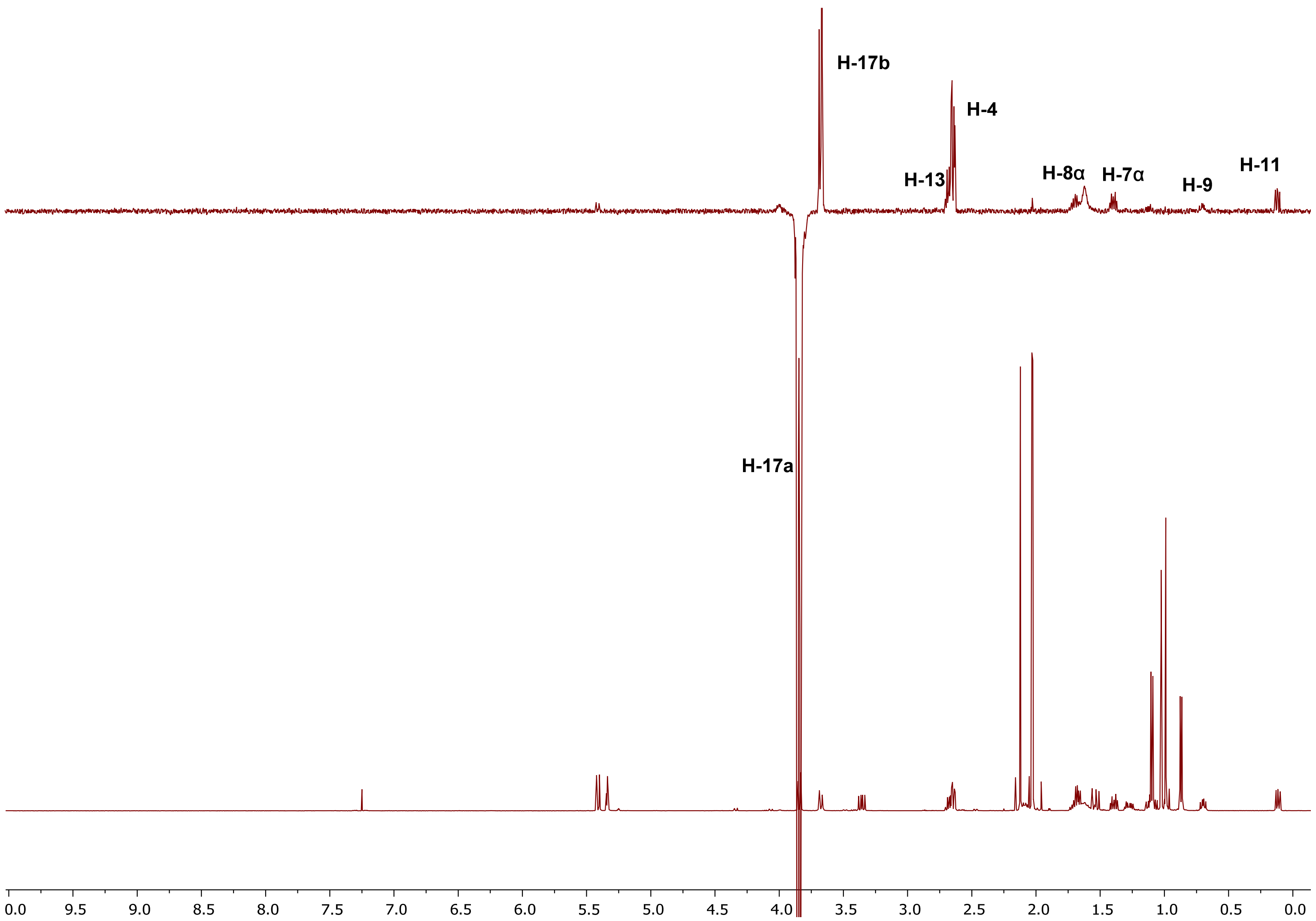

Figure S9c. 1D NOESY spectrum of compound **5**.

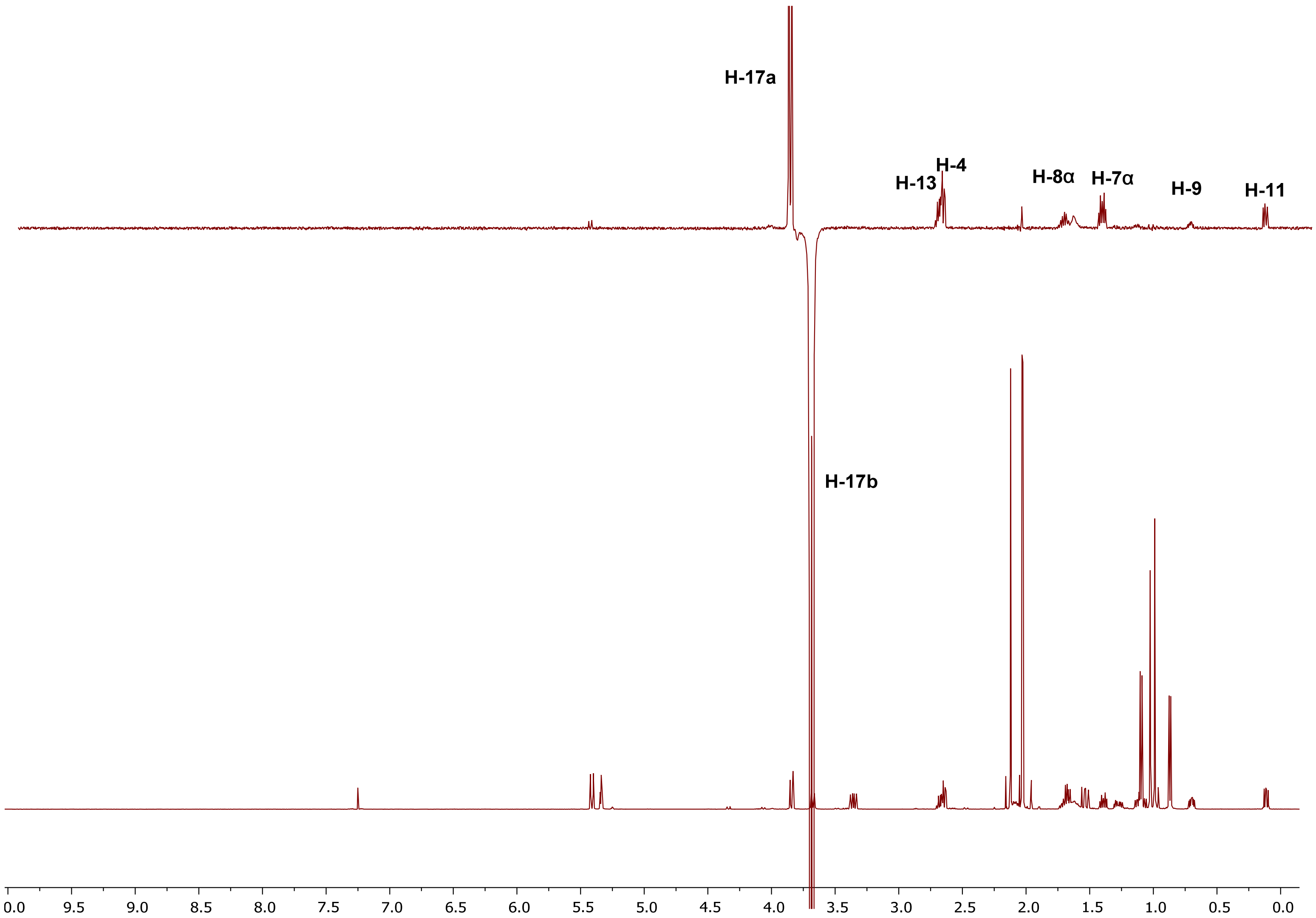

Figure S9d. 1D NOESY spectrum of compound **5**.

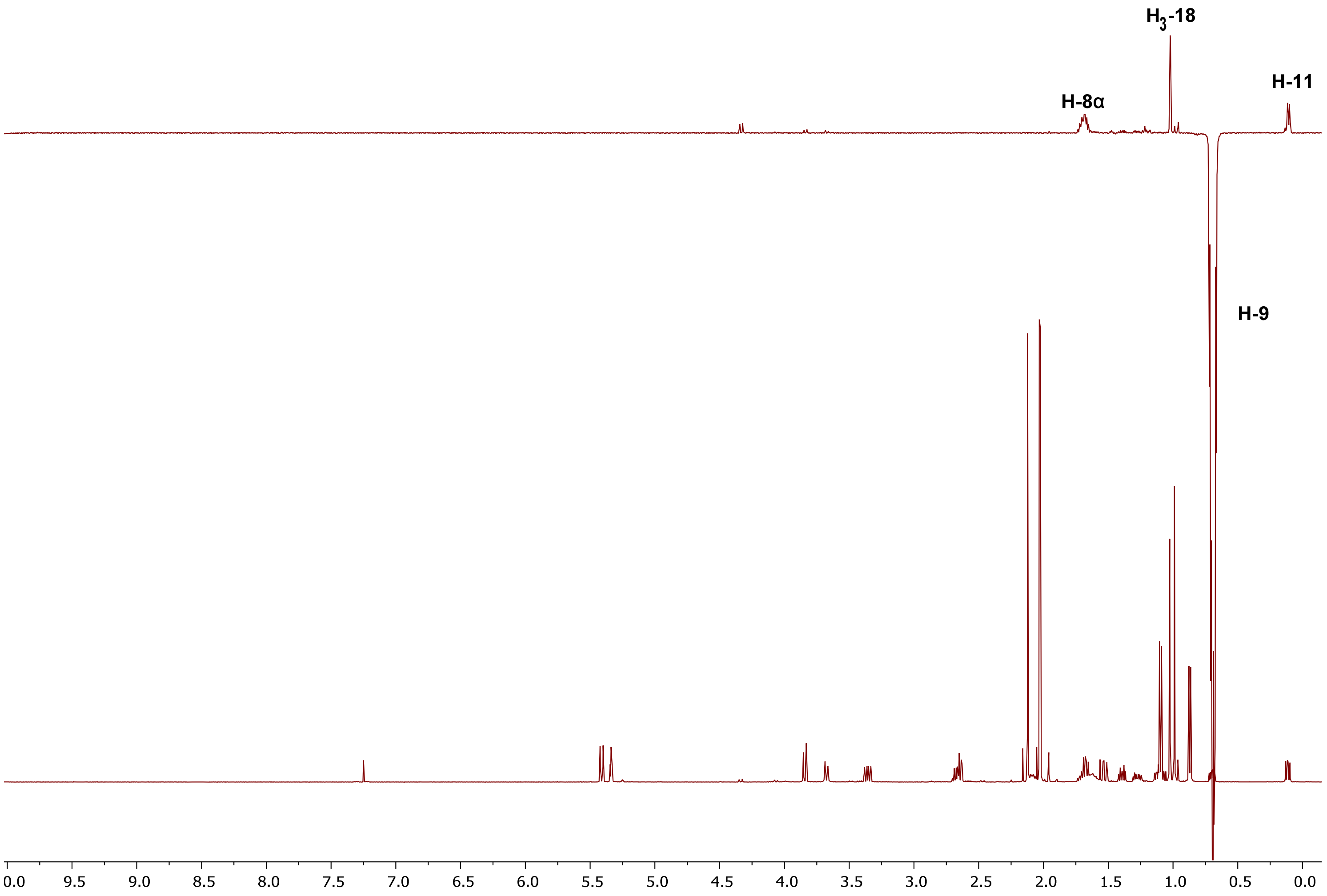

Figure S9e. 1D NOESY spectrum of compound **5**.

Monoisotopic Mass, Even Electron Ions
87 formula(e) evaluated with 2 results within limits (all results (up to 1000) for each mass)
Elements Used:
C: 0-30 H: 0-50 O: 0-15 23Na: 0-1
FEM-248 456 (4.225)

1: TOF MS ES+
1.09e+006

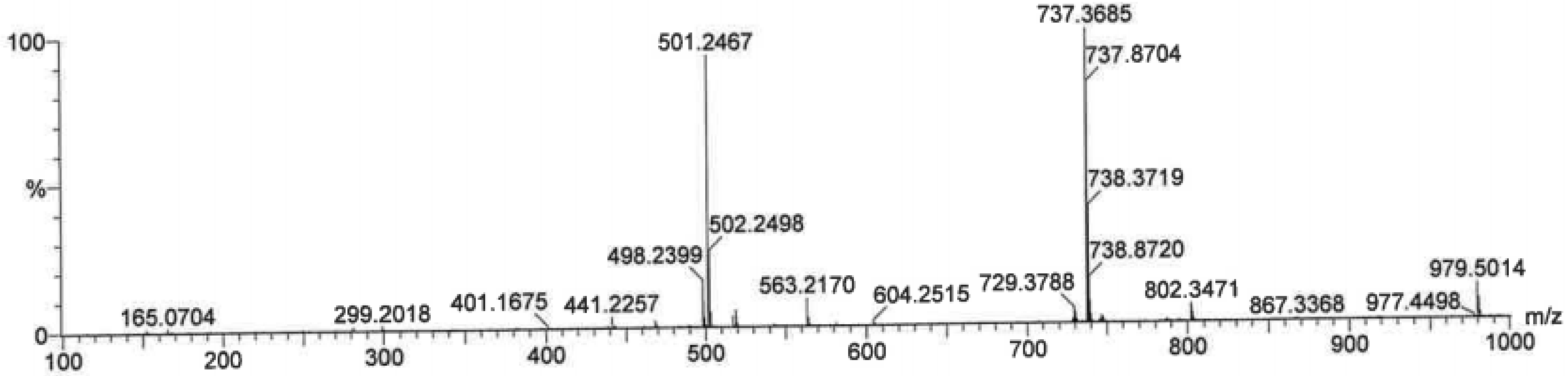

		mDa	PPM	DBE	i-FIT	Norm	Conf(%)	Formula
Minimum:				-1.5				
Maximum:		5.0	10.0	80.0				
**Mass**	**Calc. Mass**	**mDa**	**PPM**	**DBE**	**i-FIT**	**Norm**	**Conf(%)**	**Formula**
501.2467	501.2464	0.3	0.6	7.5	1351.8	0.002	99.75	C26 H38 O8 23Na
	501.2488	-2.1	-4.2	10.5	1357.8	6.000	0.25	C28 H37 O8

Figure S10. HRMS of compound **5**.

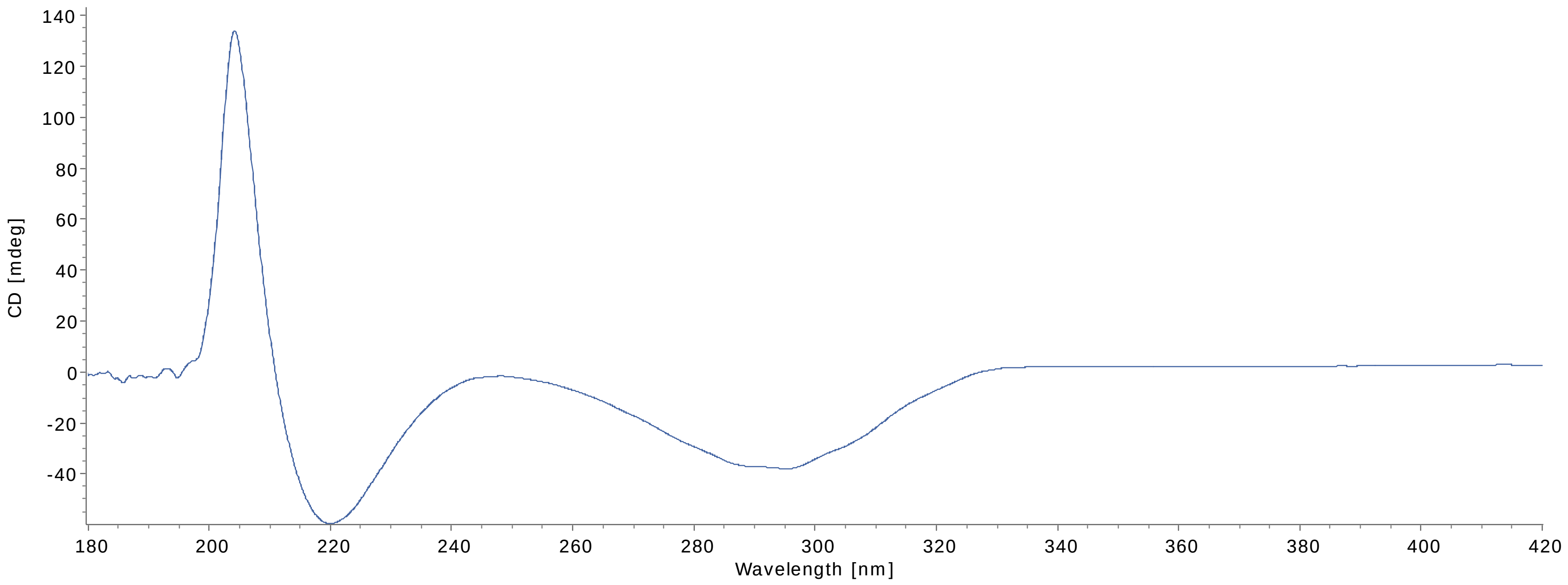

Figure S11. ECD of compound **5**.

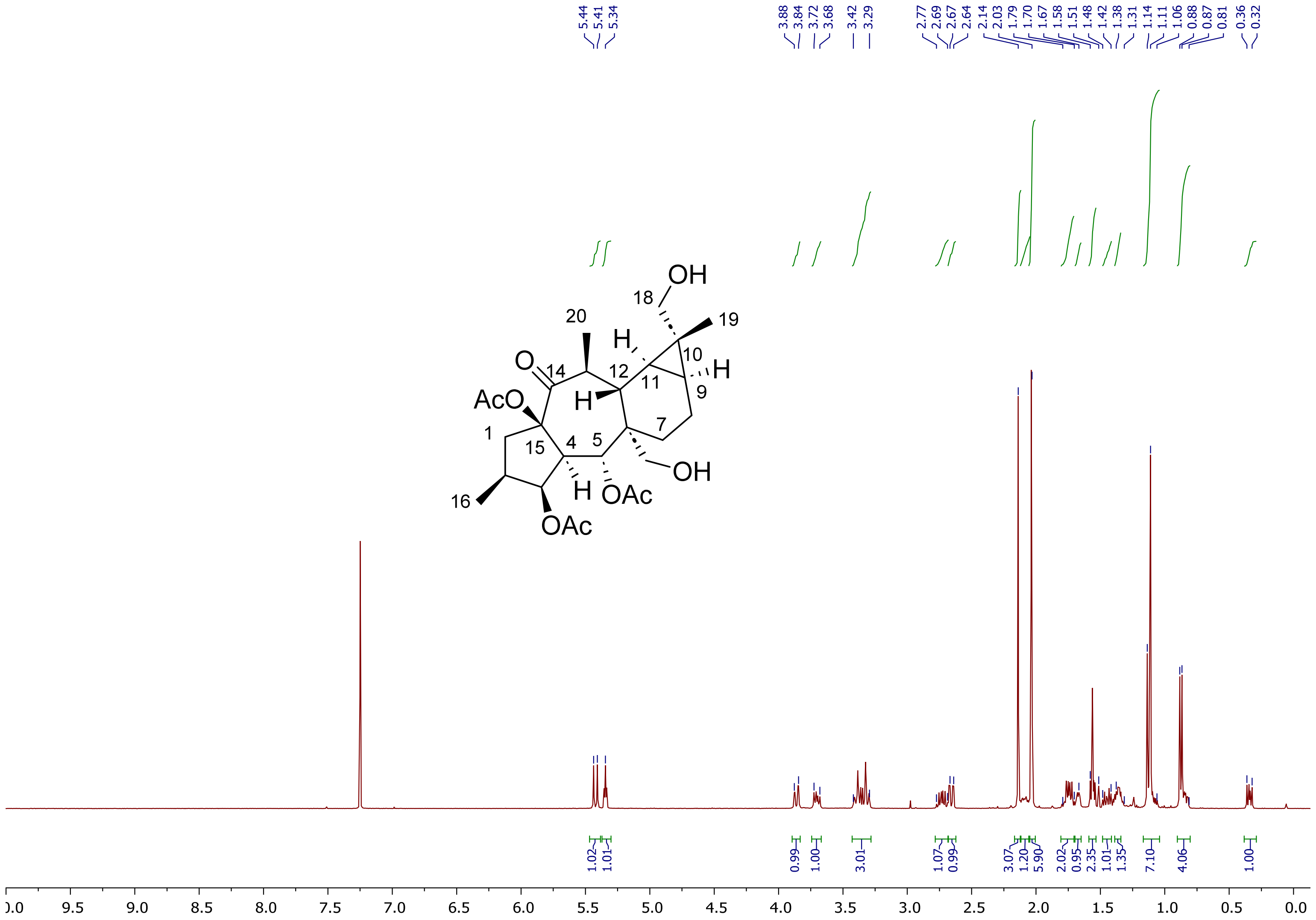

Figure S12. ^{1}H NMR spectrum (400 MHz) of compound **6** in $CDCl_3$.

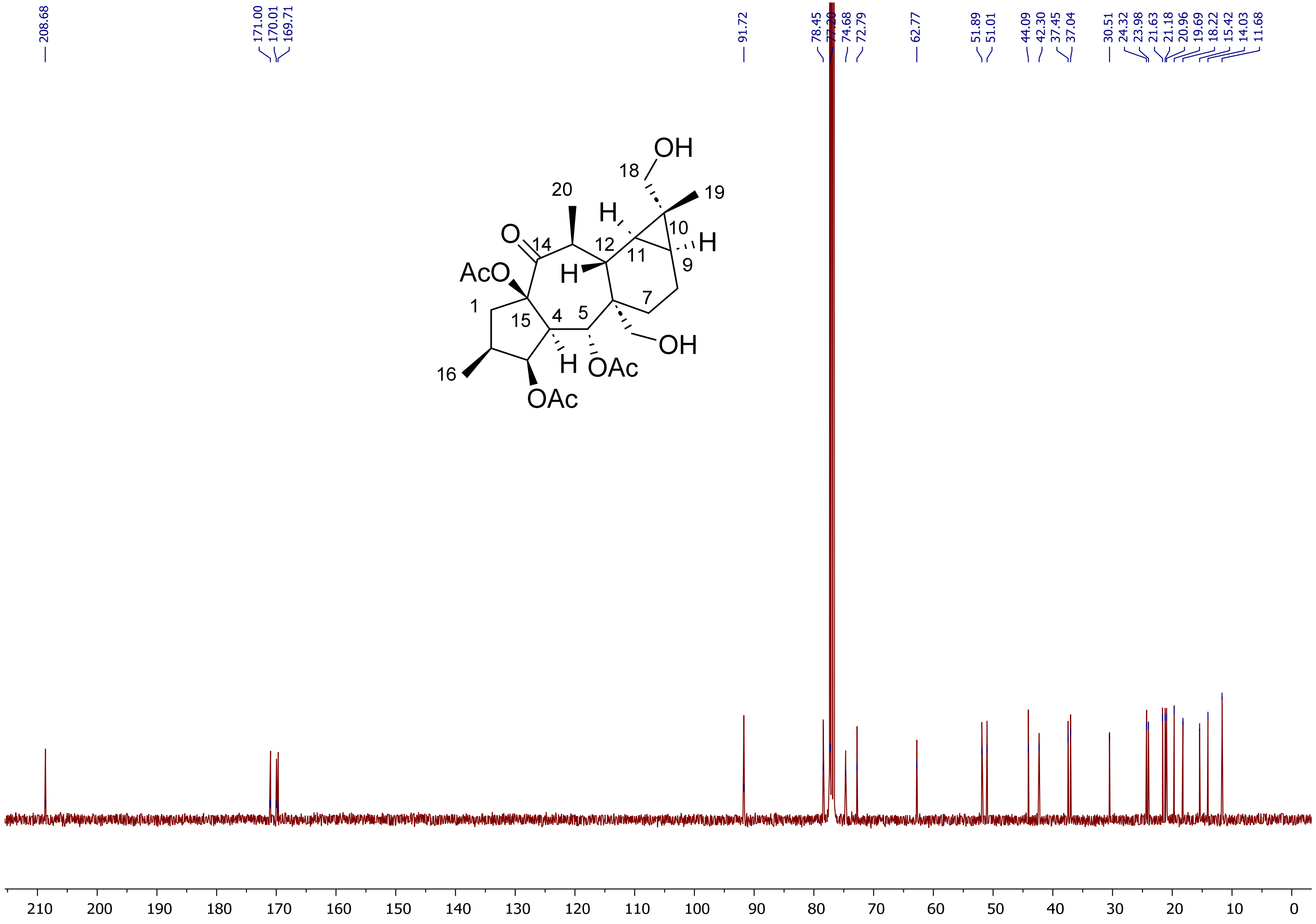

Figure S13. ^{13}C NMR spectrum (100 MHz) of compound **6** in $CDCl_3$.

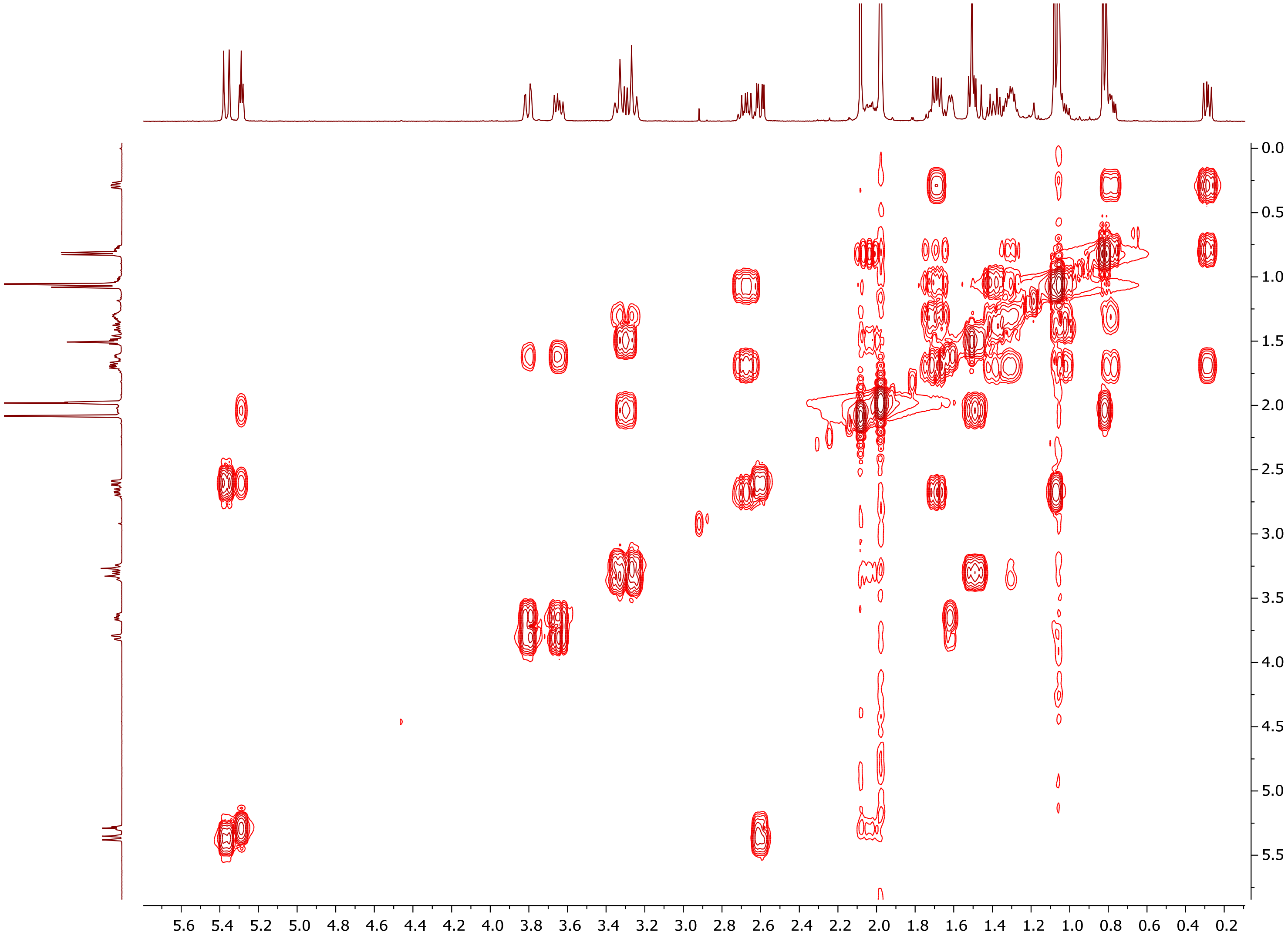

Figure S14. gCOSY spectrum of compound **6**.

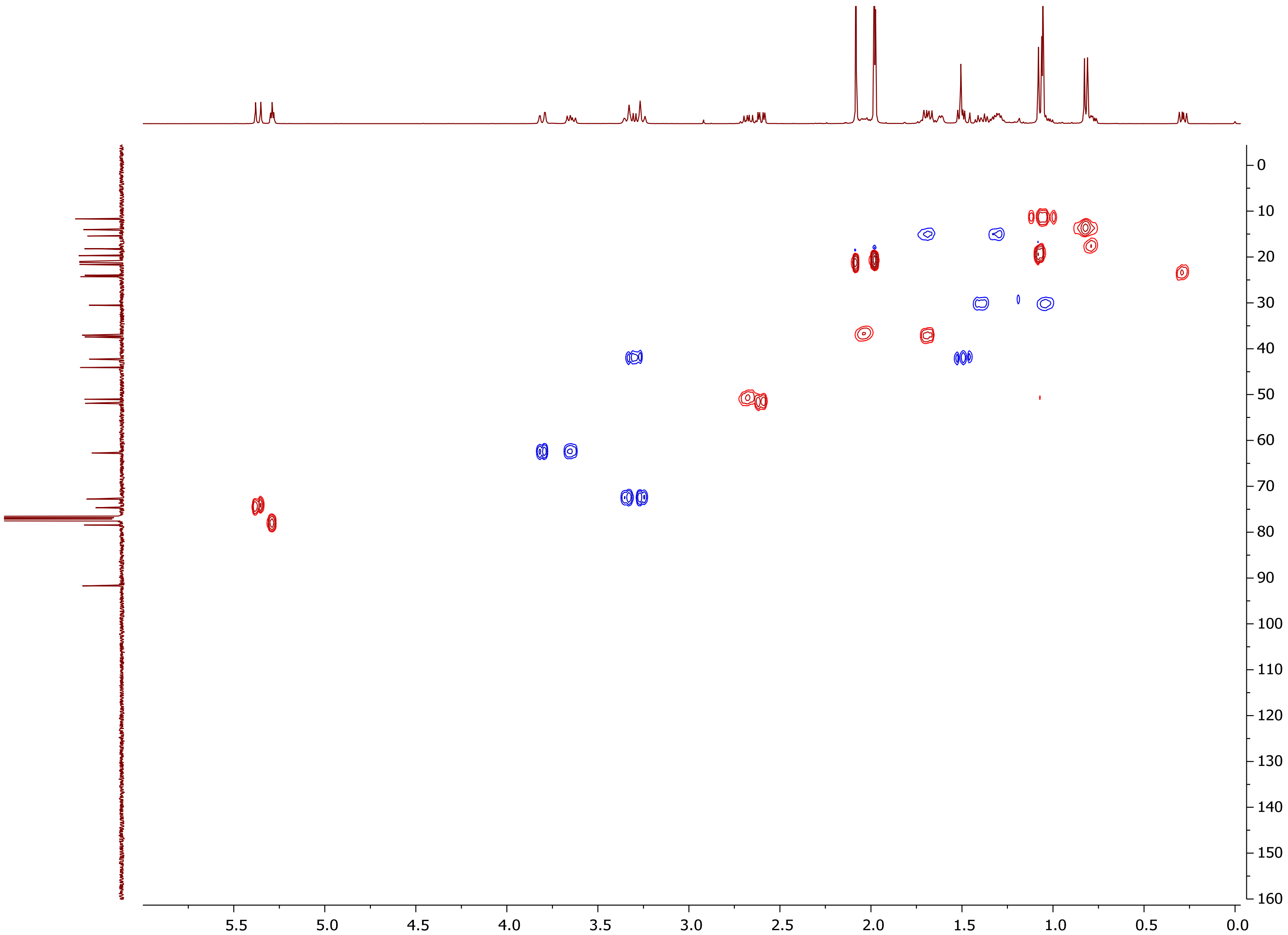

Figure S15. gHSQC spectrum of compound **6**.

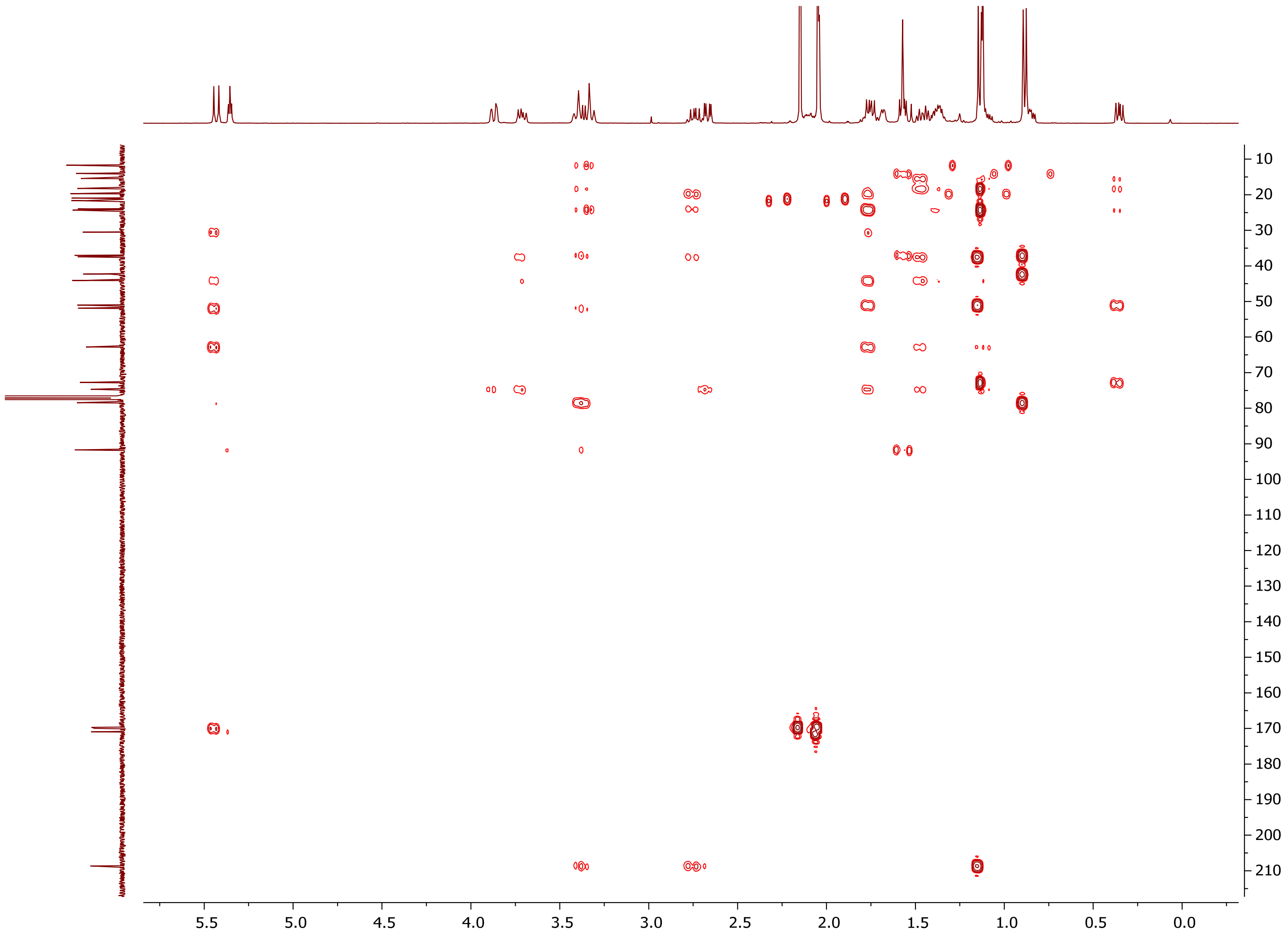

Figure S16. gHMBC spectrum of compound **6**.

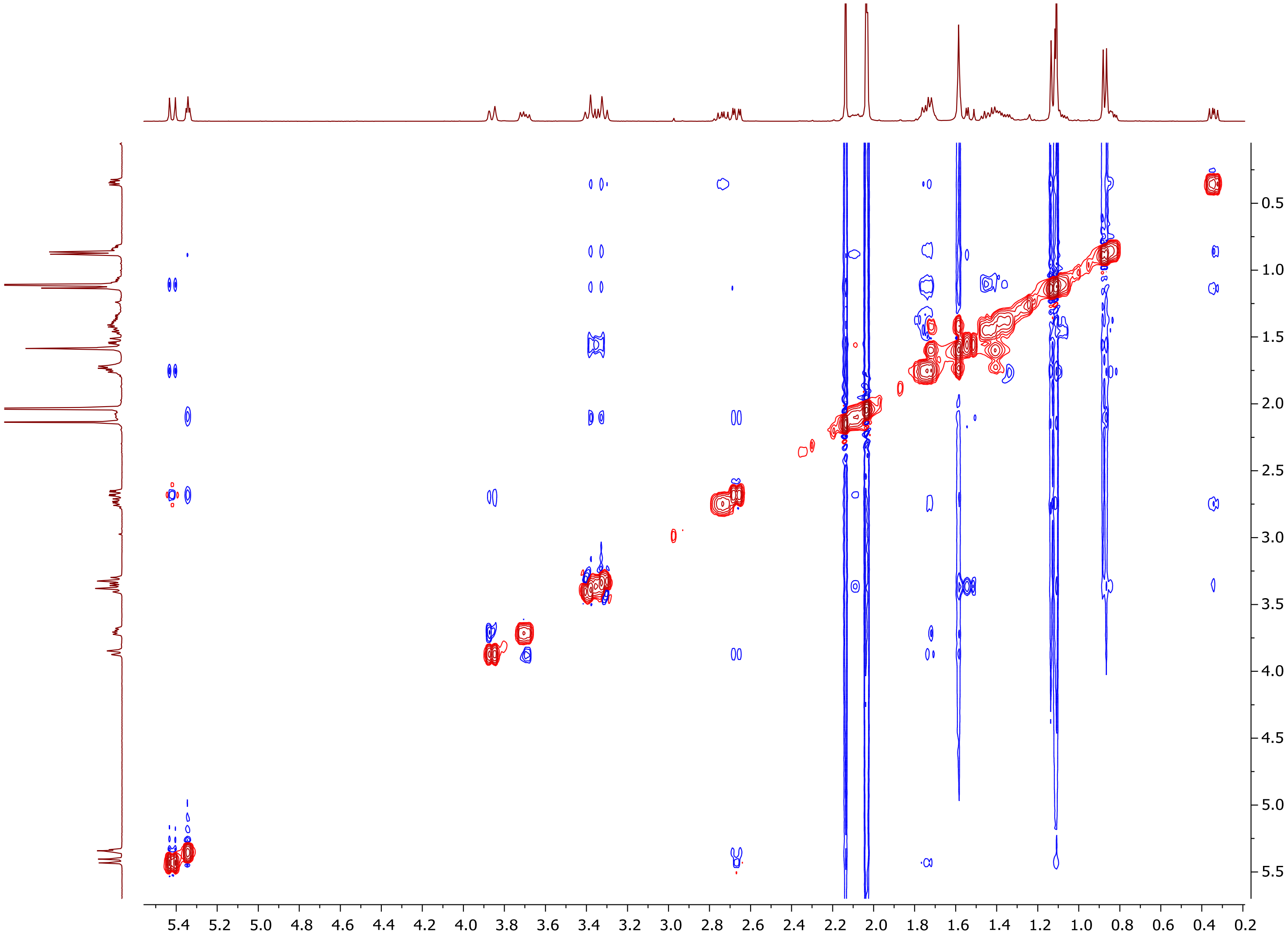

Figure S17. 2D NOESY spectrum of compound **6**.

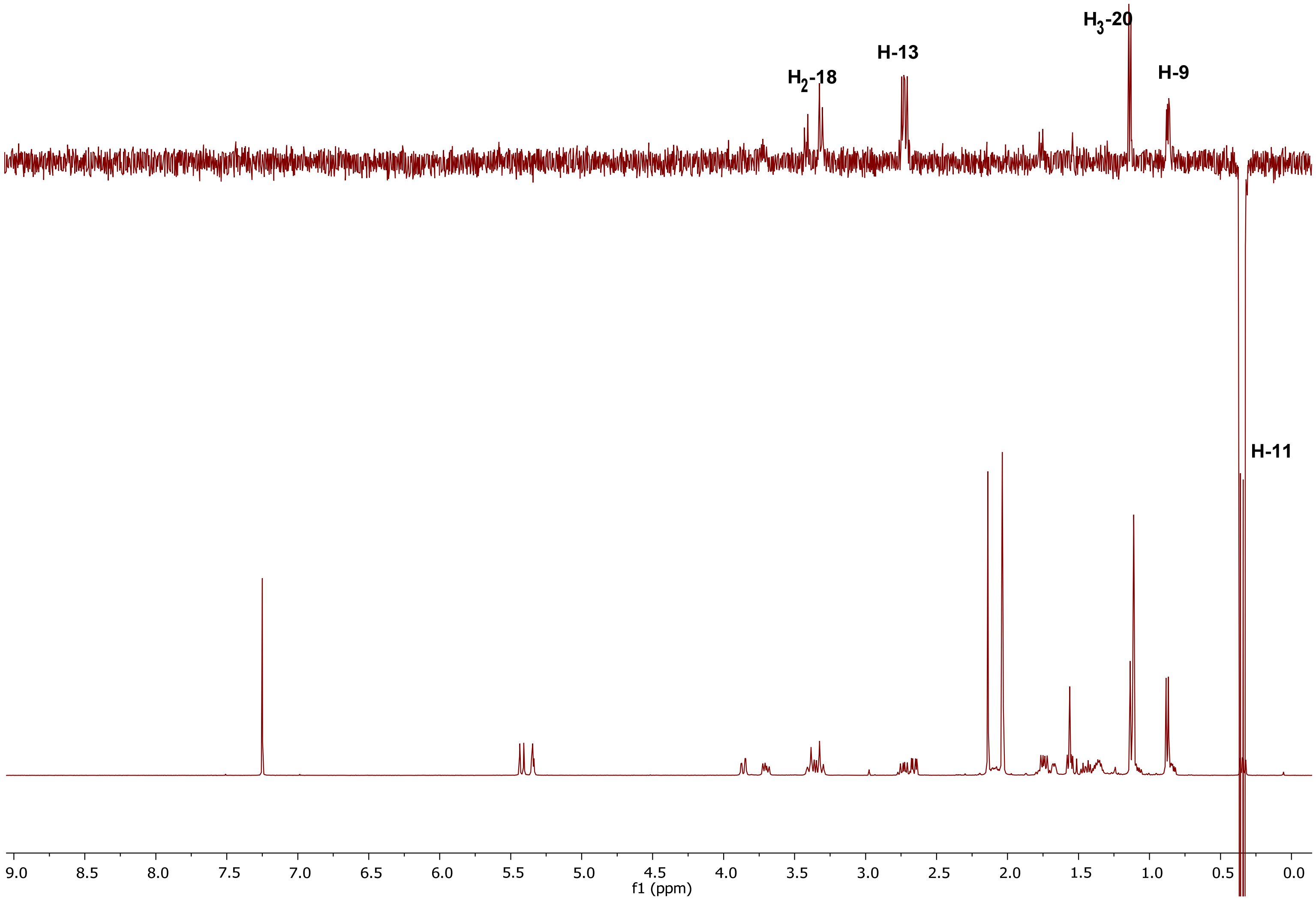

Figure S18a. 1D NOESY spectrum of compound **6**.

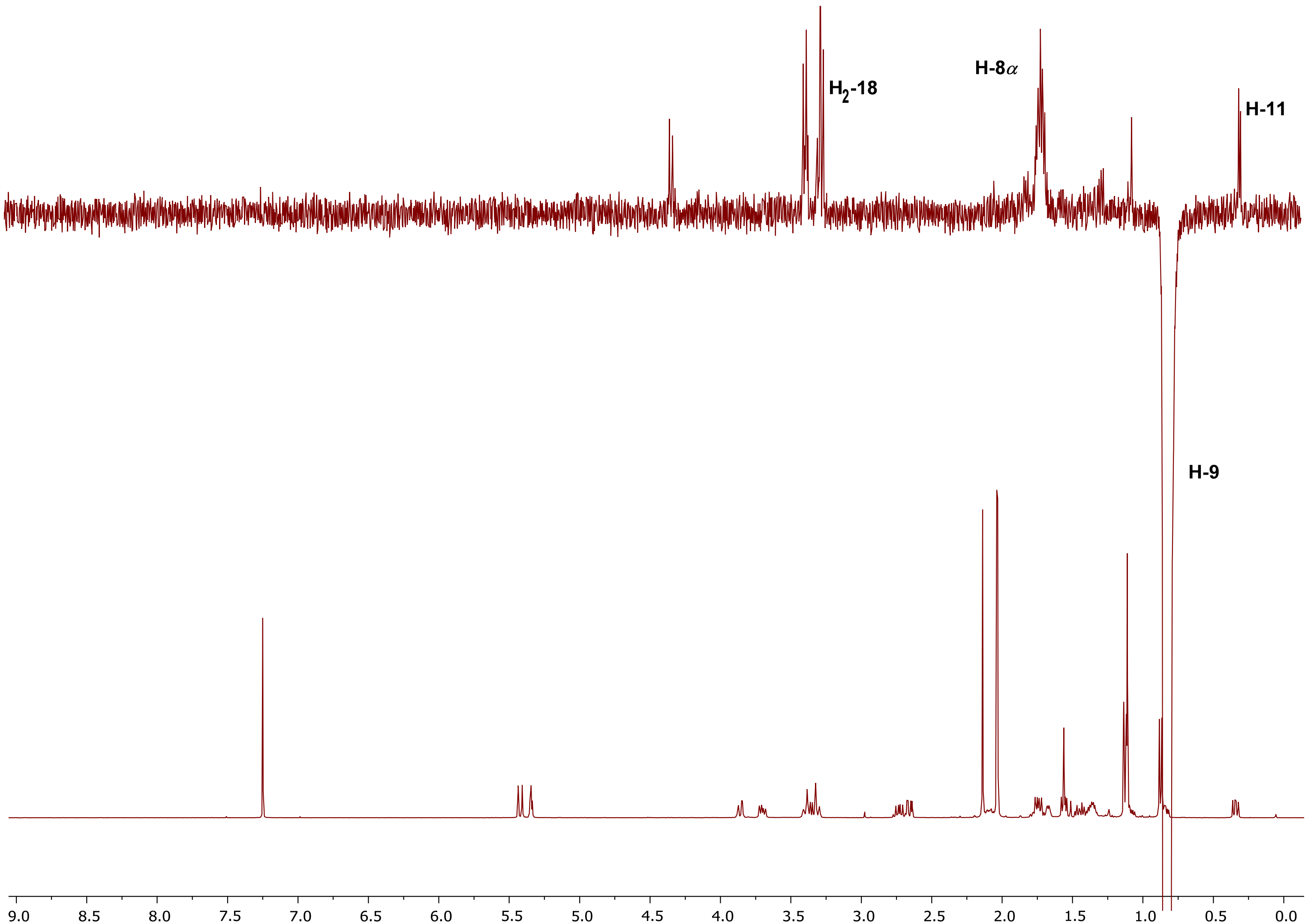

Figure S18b. 1D NOESY spectrum of compound **6**.

Monoisotopic Mass, Even Electron Ions
82 formula(e) evaluated with 2 results within limits (all results (up to 1000) for each mass)
Elements Used:
C: 0-30 H: 0-50 O: 0-15 23Na: 0-1
FEM-252 320 (2.967)

1: TOF MS ES+
3.69e+005

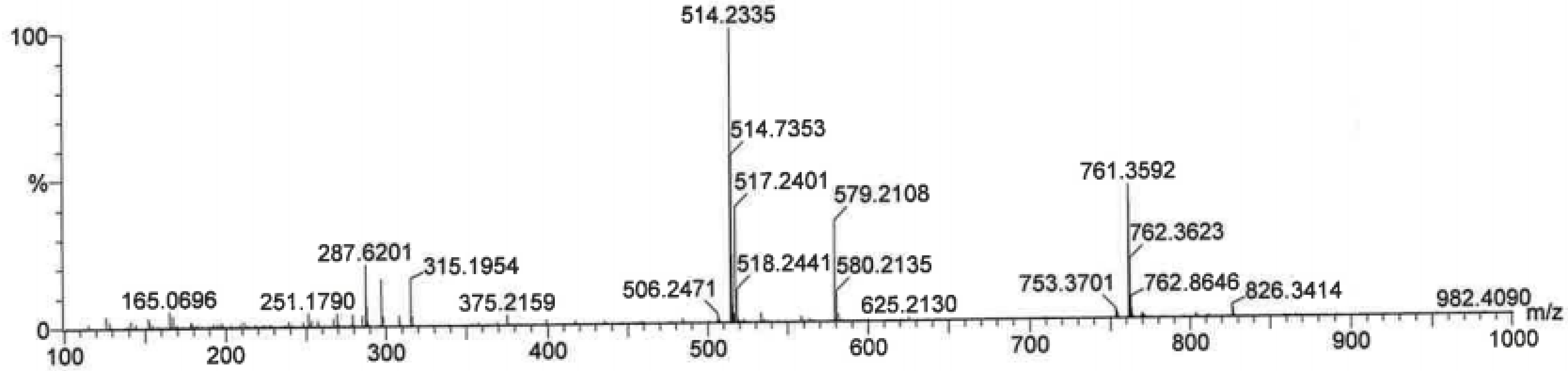

Minimum:				-1.5				
Maximum:		5.0	10.0	80.0				
Mass	Calc. Mass	mDa	PPM	DBE	i-FIT	Norm	Conf(%)	Formula
517.2401	517.2414	-1.3	-2.5	7.5	939.2	0.311	73.29	C26 H38 O9 23Na
	517.2438	-3.7	-7.2	10.5	940.2	1.320	26.71	C28 H37 O9

Figure S19. HRMS of compound **6**.

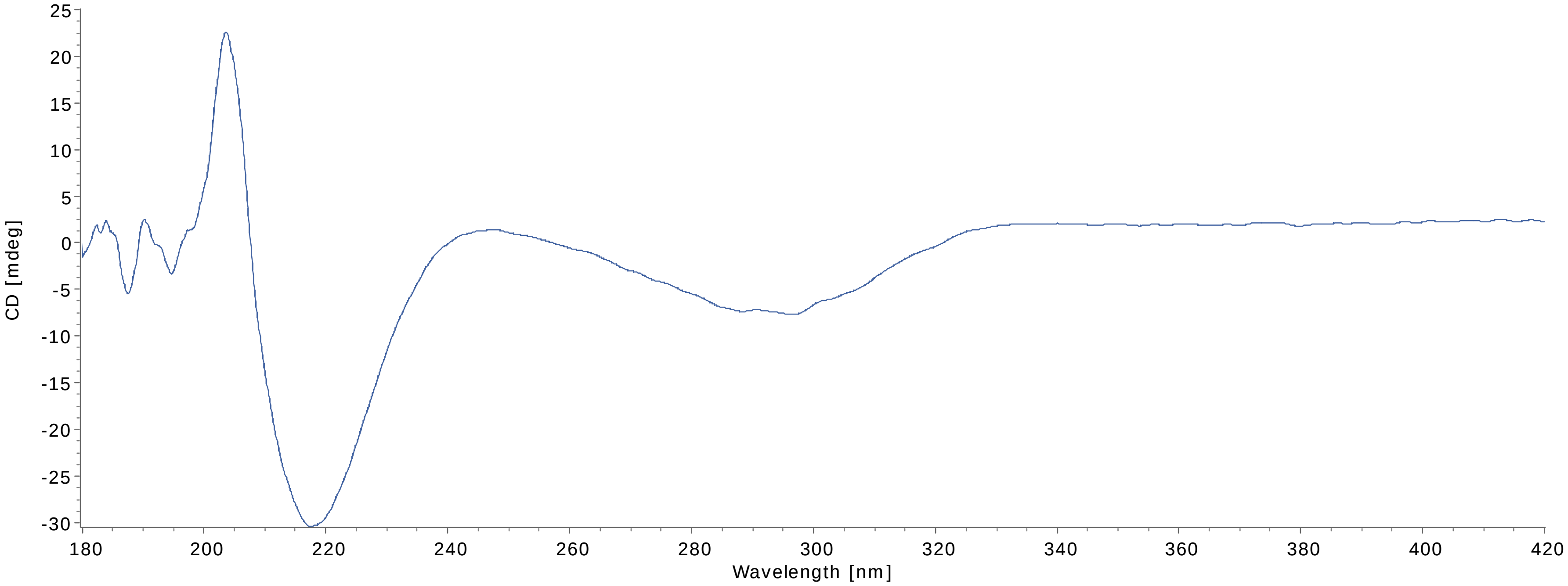

Figure S20. ECD of compound **6**.

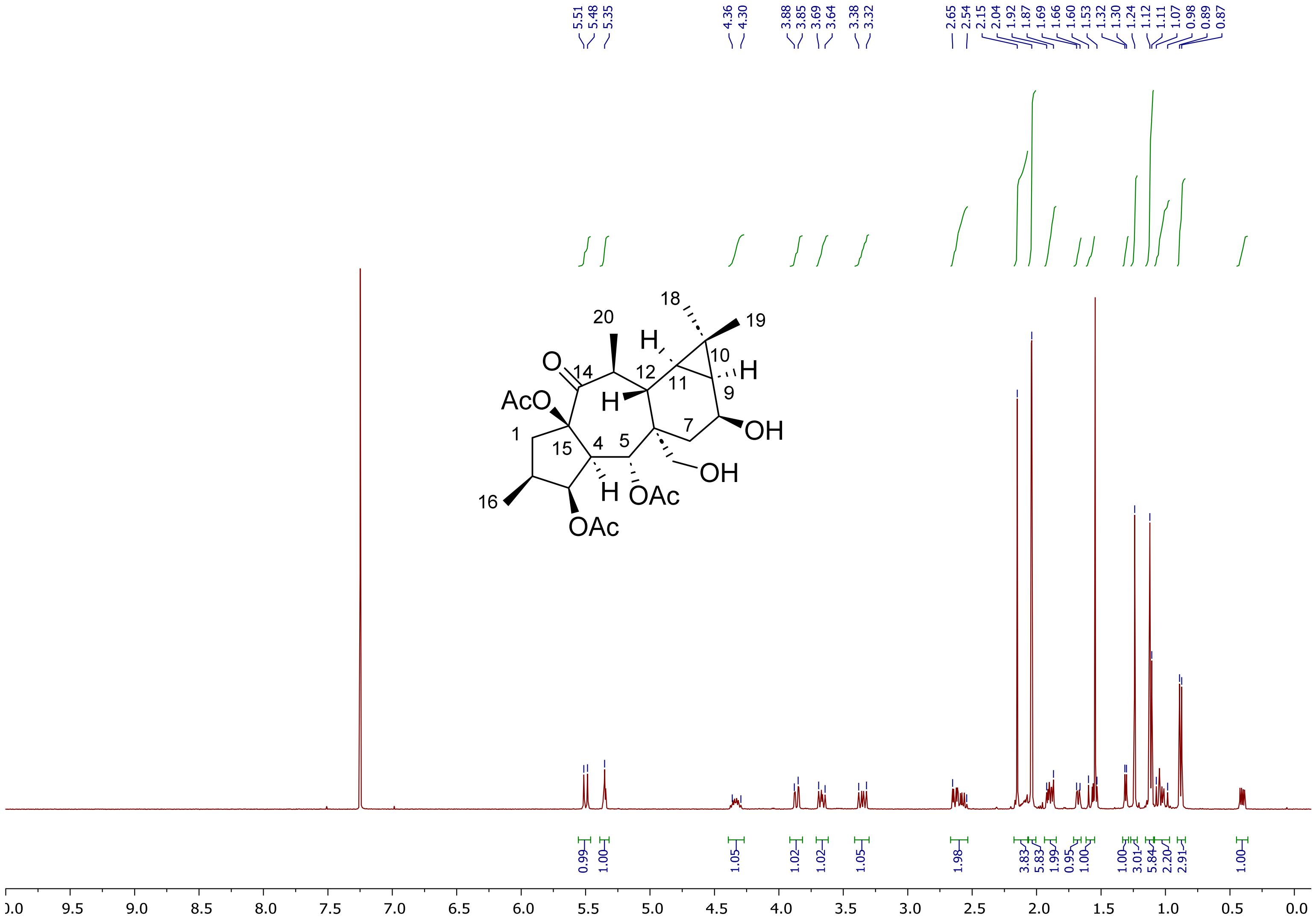

Figure S21. ^{1}H NMR spectrum (400 MHz) of compound **7** in $CDCl_3$.

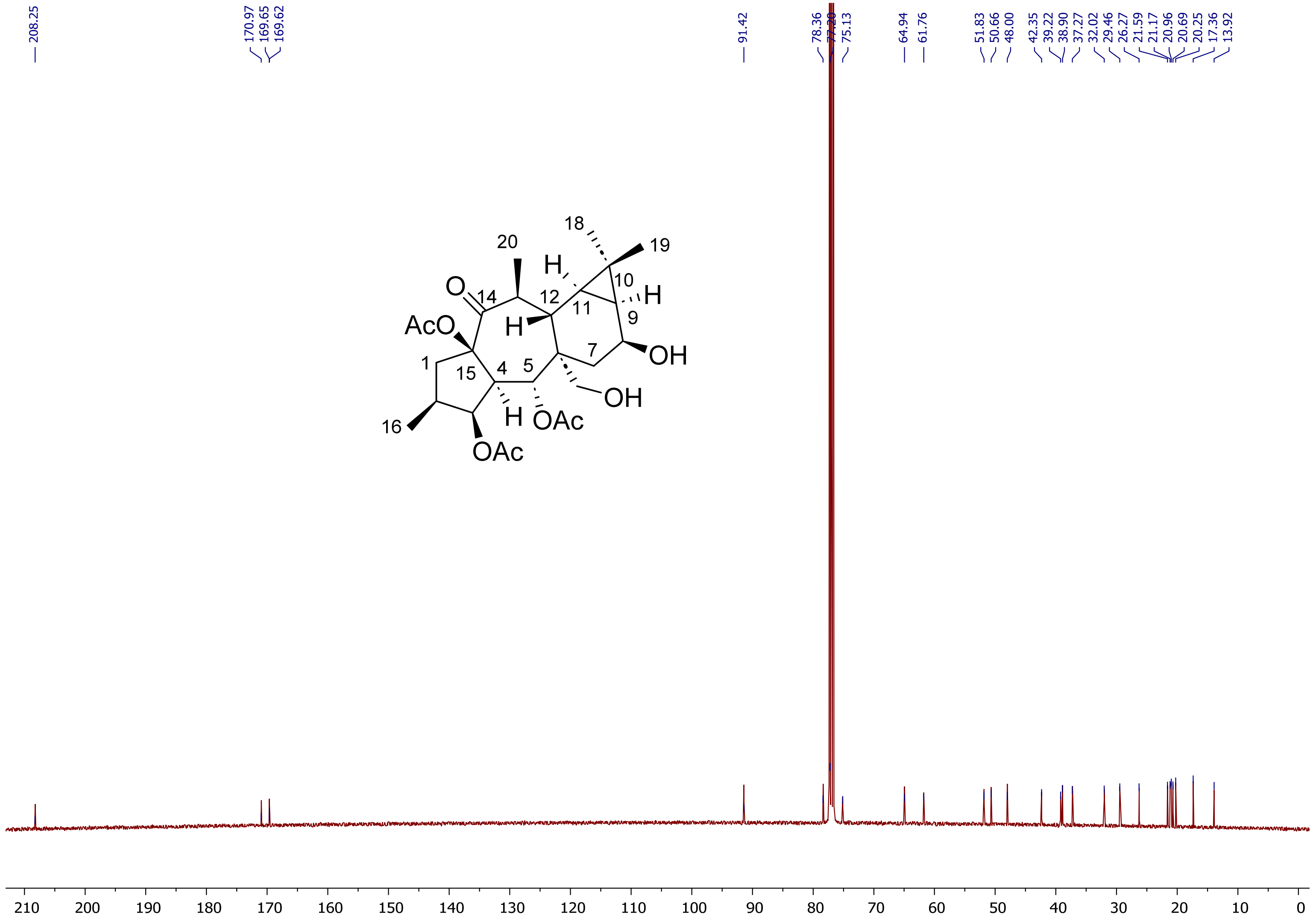

Figure S22. ^{13}C NMR spectrum (100 MHz) of compound **7** in $CDCl_3$.

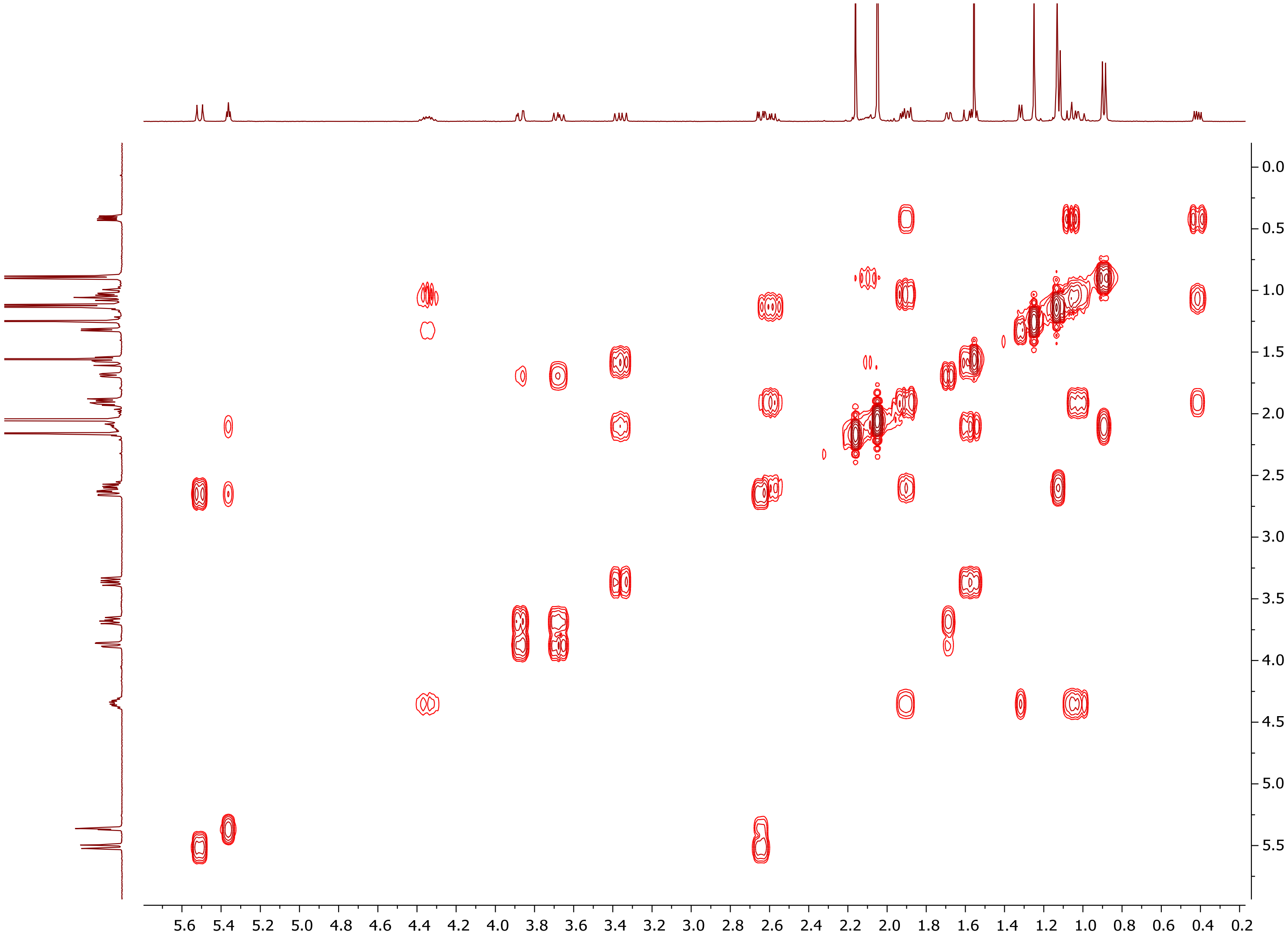

Figure S23. gCOSY spectrum of compound **7**.

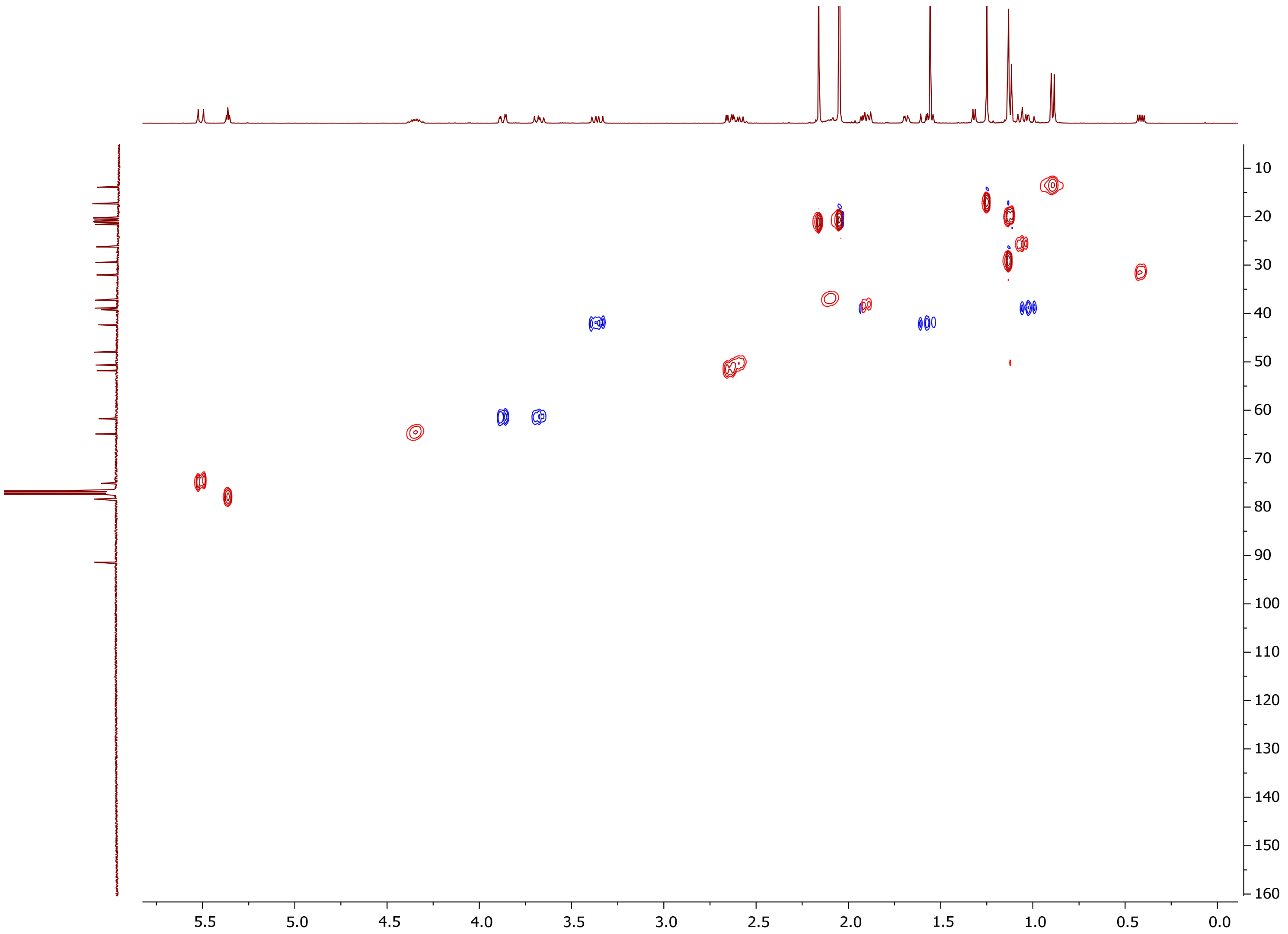

Figure S24. gHSQC spectrum of compound **7**.

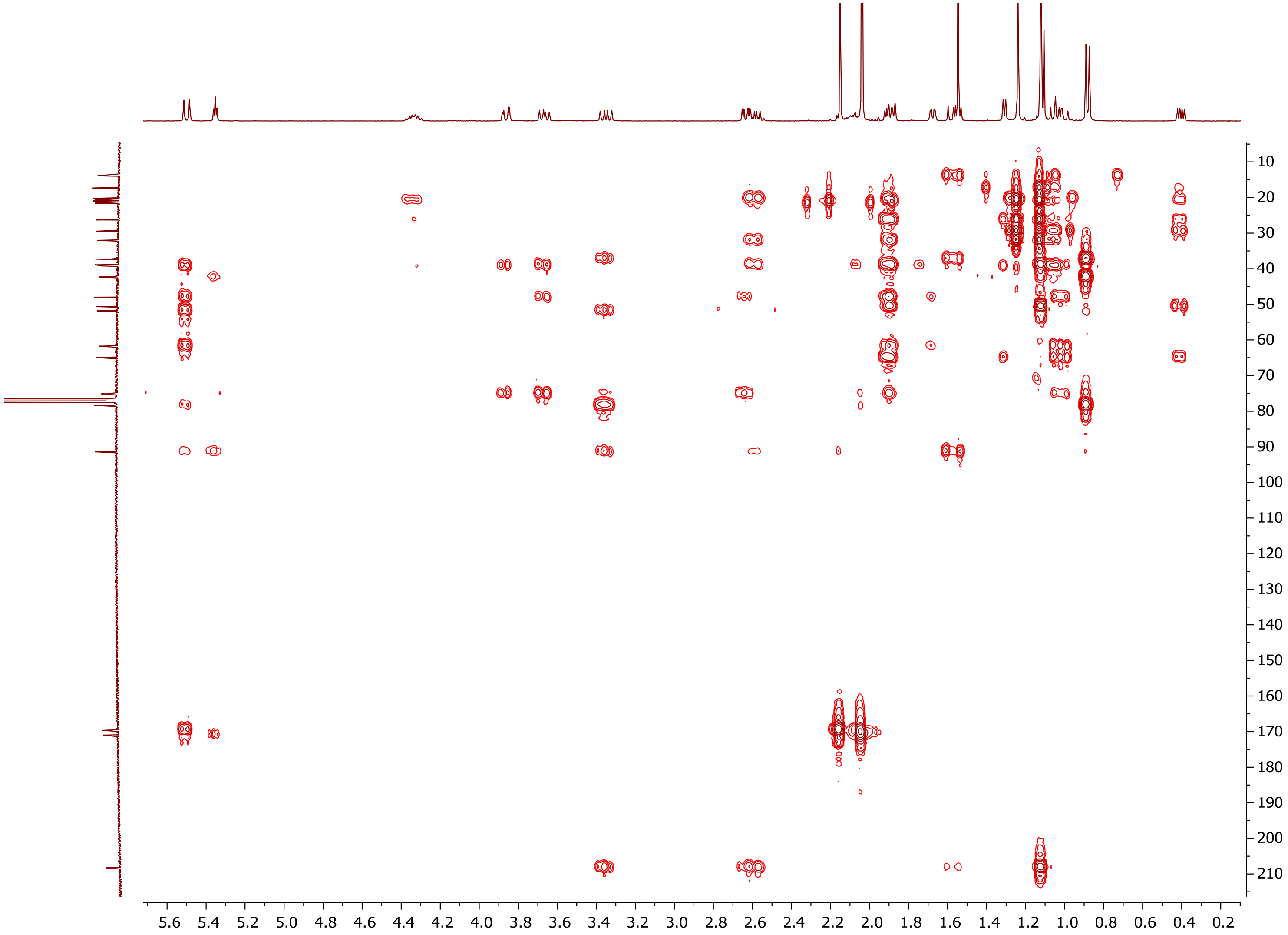

Figure S25. gHMBC spectrum of compound **7**.

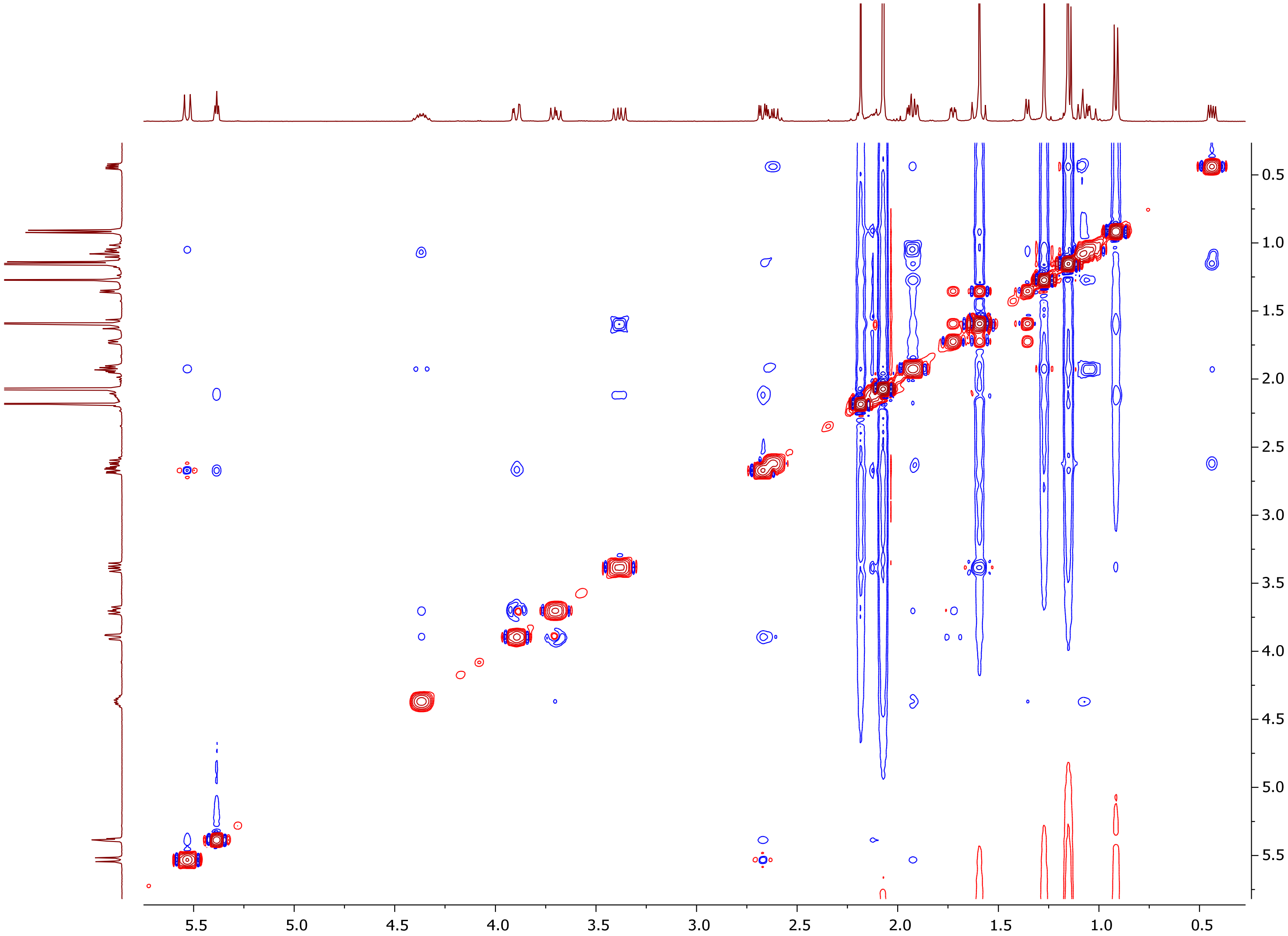

Figure S26. 2D NOESY spectrum of compound **7**.

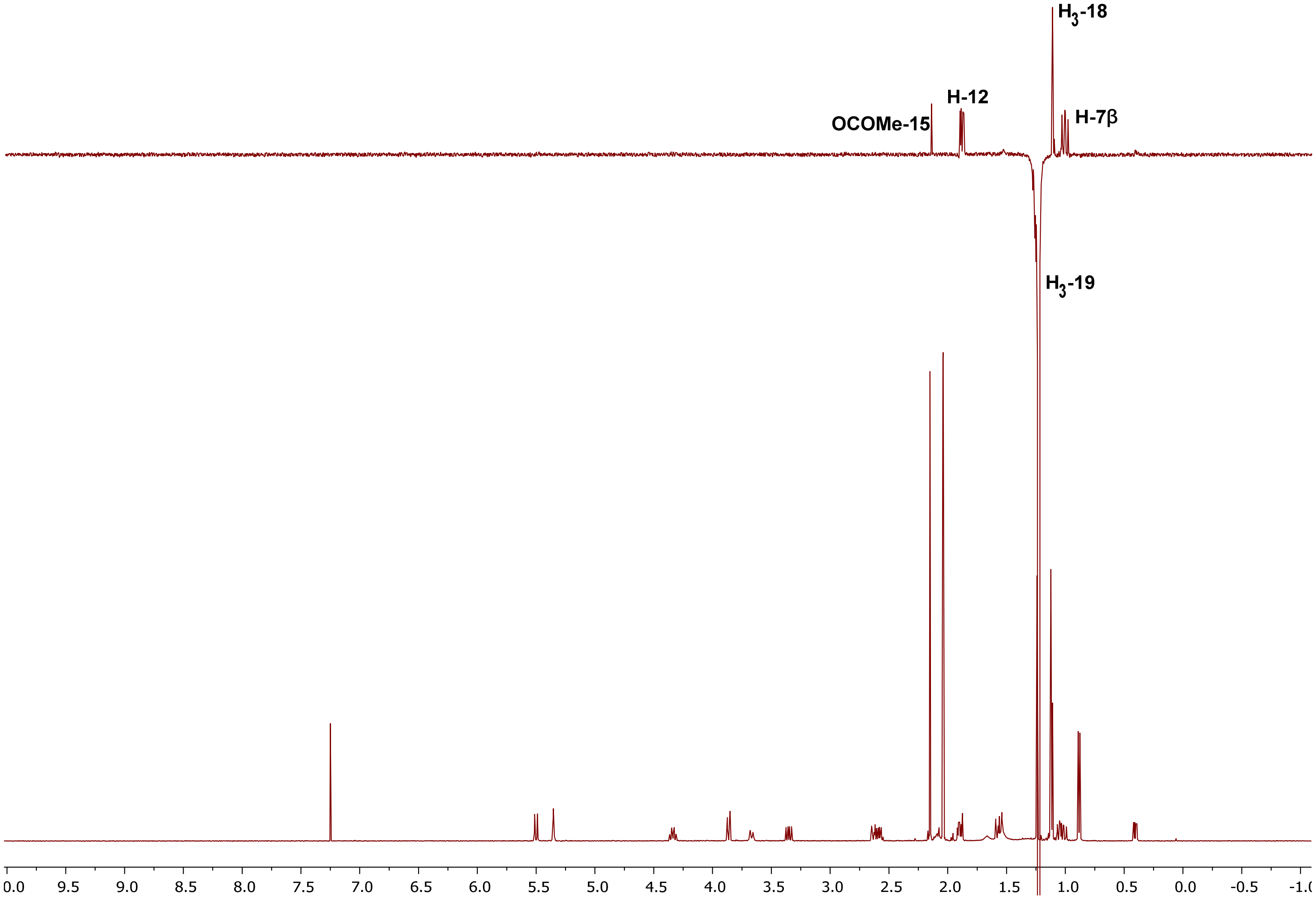

Figure S27a. 1D NOESY spectrum of compound **7**.

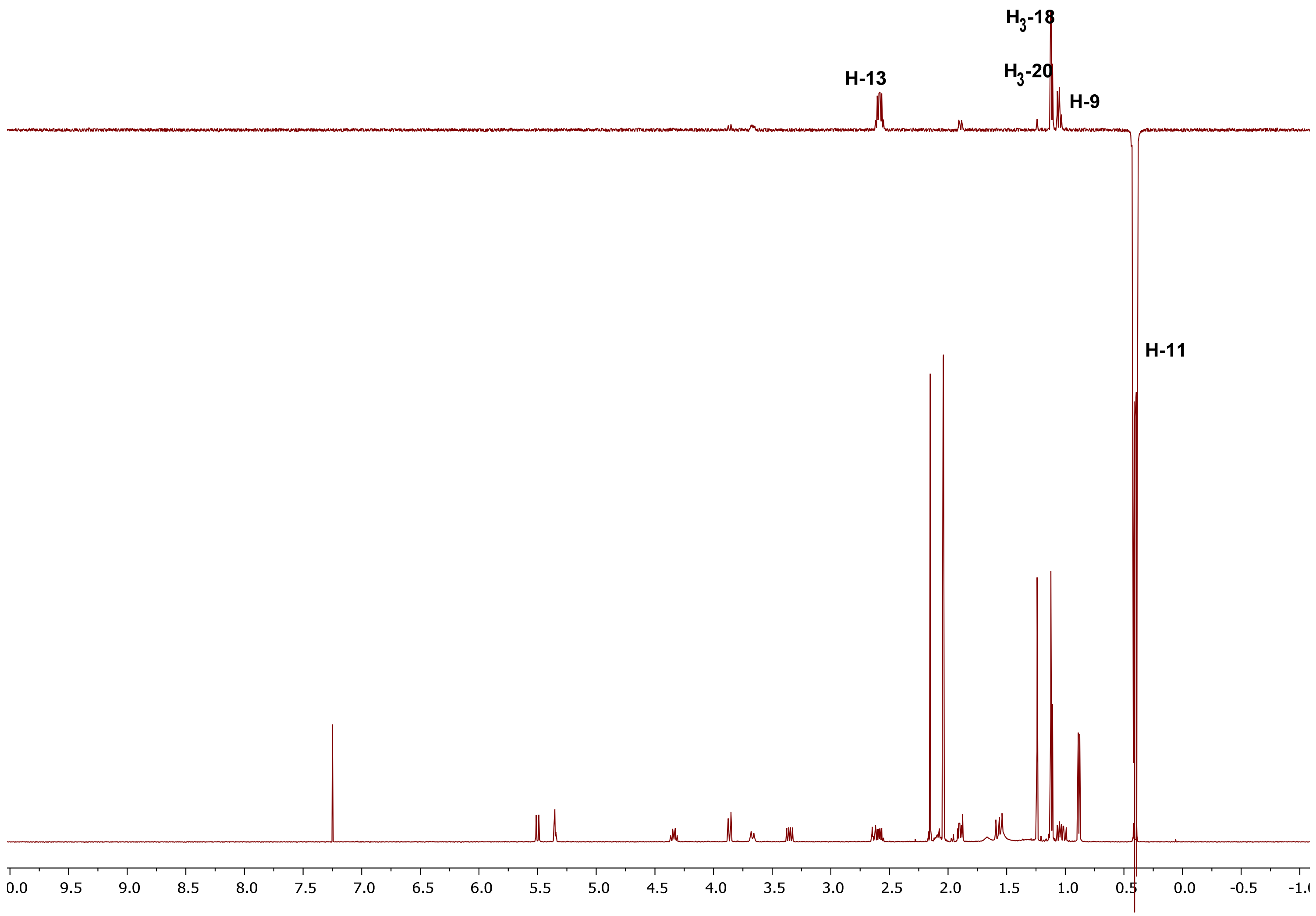

Figure S27b. 1D NOESY spectrum of compound **7**.

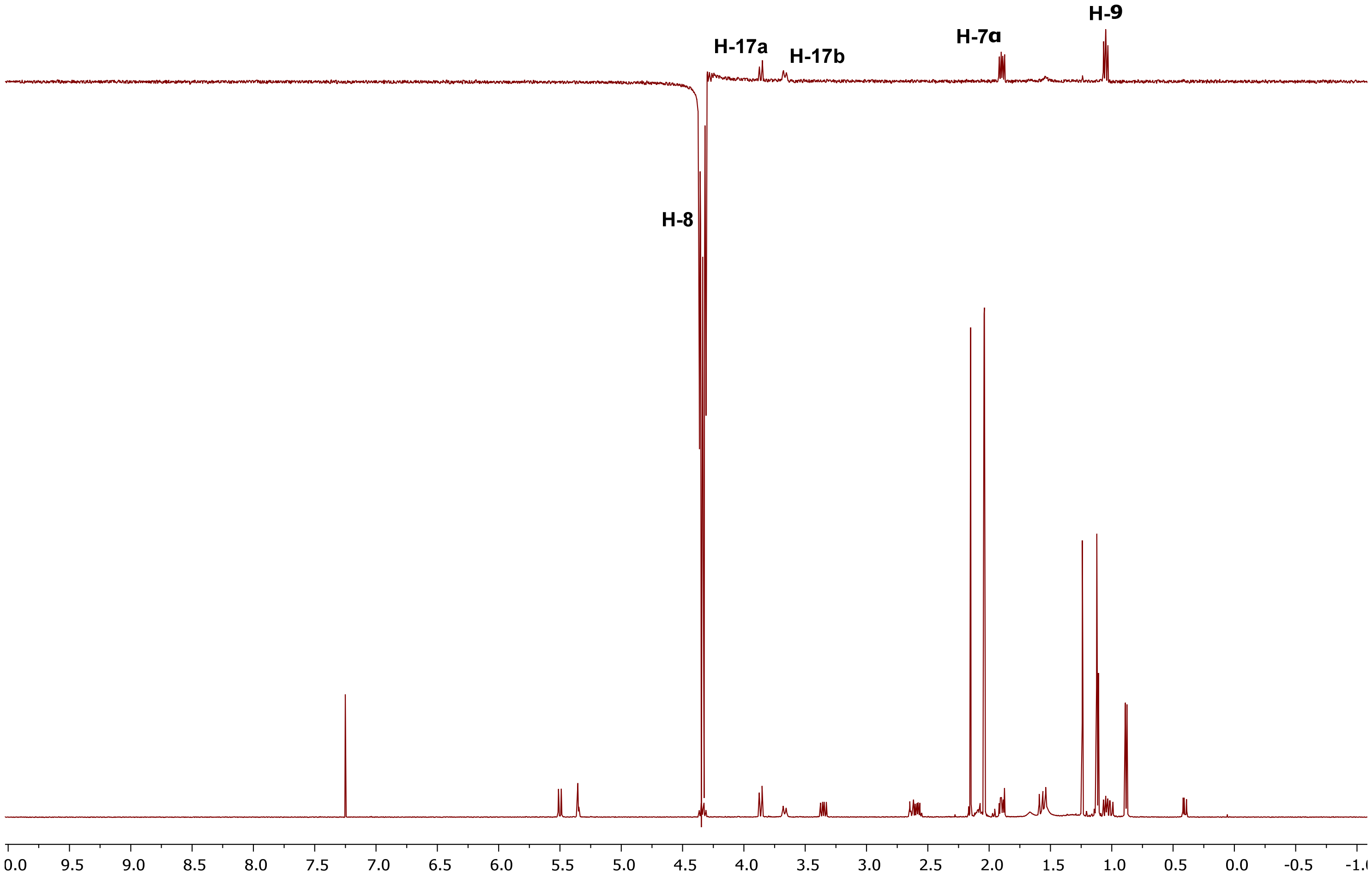

Figure S27c. 1D NOESY spectrum of compound **7**.

Monoisotopic Mass, Even Electron Ions
82 formula(e) evaluated with 2 results within limits (all results (up to 1000) for each mass)
Elements Used:
C: 0-30 H: 0-50 O: 0-15 23Na: 0-1
FEM-250 352 (3.264)

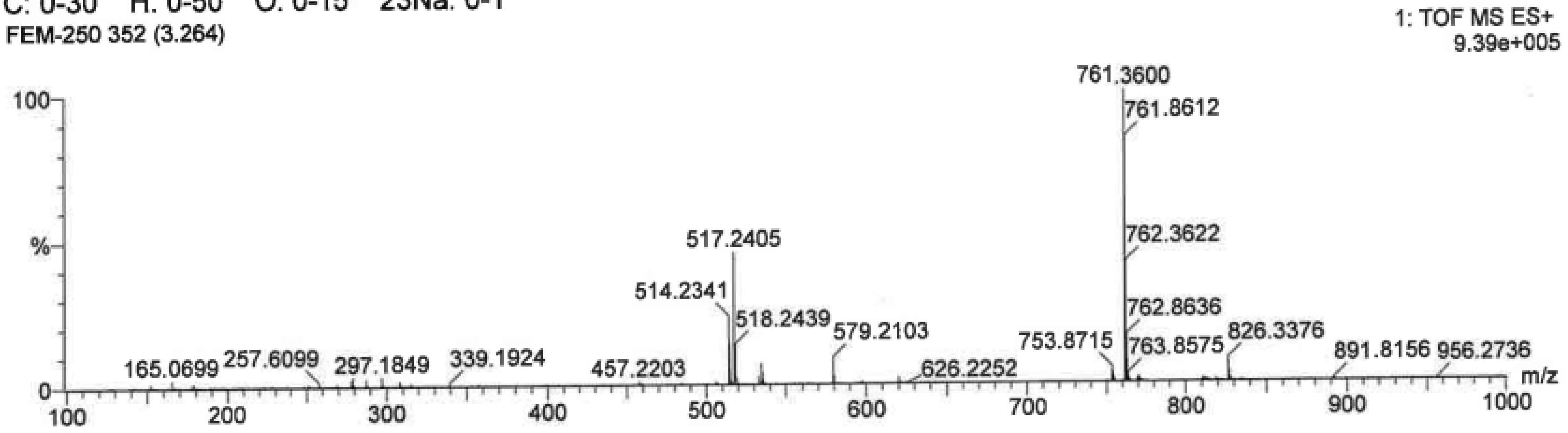

	Mass	Calc. Mass	mDa	PPM	DBE	i-FIT	Norm	Conf(%)	Formula
Minimum:					-1.5				
Maximum:			5.0	10.0	80.0				
	517.2405	517.2414	-0.9	-1.7	7.5	1177.5	0.057	94.46	C26 H38 O9 23Na
		517.2438	-3.3	-6.4	10.5	1180.4	2.893	5.54	C28 H37 O9

Figure S28. HRMS of compound **7**.

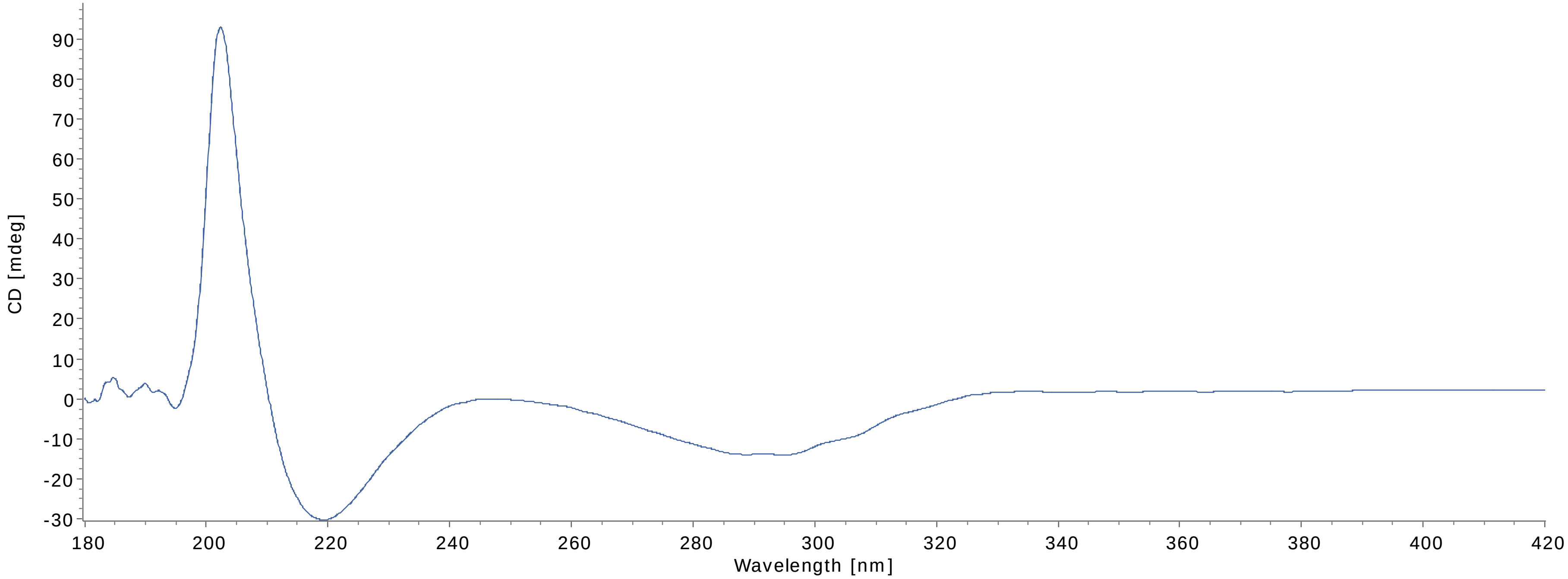

Figure S29. ECD of compound **7**.
